# Understanding patterns of variant emergence and spread in an ongoing epidemic

**DOI:** 10.64898/2026.03.27.26349560

**Published:** 2026-03-30

**Authors:** Anjalika Nande, Michael Z. Levy, Alison L. Hill

**Affiliations:** 1 Institute for Computational Medicine, Johns Hopkins University, Baltimore, MD, USA; 2 Nuffield Department of Primary Care Health Sciences, University of Oxford, Oxford, UK; 3 Department of Biostatistics, Epidemiology and Informatics, University of Pennsylvania, Philadelphia, PA, USA; 4 Department of Ecology & Evolutionary Biology, University of Toronto, Toronto, ON, Canada

## Abstract

The COVID-19 pandemic saw successive emergence and global spread of novel viral variants, exhibiting enhanced transmissibility or evasion of immunity. While the genotypic and phenotypic basis of SARS-CoV-2 variants have been extensively characterized, the evolutionary factors governing their patterns of emergence are less well understood. In this study we systematically investigated how the invasion dynamics of viral variants depend on variant phenotype (increased transmissibility or immune evasion), source (local evolution vs importation), the timing of introduction, the distribution of population susceptibility, and the contact network structure. Using a stochastic multi-strain epidemic model, we find that strains with only a transmission advantage are more likely to emerge earlier in the epidemic, and rapidly and predictably dominate the viral population. In contrast, immune-escape variants tend to linger at low prevalence for extended time periods after emergence, avoiding detection, until a critical amount of immunity has built up in the population and they begin to rapidly outcompete existing strains. We find that two common features of realistic human contact networks—heterogeneity in contacts (overdispersion) and clustering—lead to more punctuated evolutionary dynamics. This work provides insight into past dynamics of SARS-CoV-2 variants and can help define planning scenarios for future epidemic modeling efforts.

## Introduction

A defining feature of the COVID-19 pandemic has been the repeated emergence of variants of concern that have compromised public health measures and contributed to successive waves of disease over several years [1]. SARS-CoV-2 variants like Alpha (B.1.1.7 [2]), Delta (B.1.617.2 [3]), and Omicron (B.1.1.529 [4]) were first detected in specific locations before eventually spreading globally and completely replacing existing strains. While the genetic basis of these variants and their phenotypic effects on cellular infectivity, antibody neutralization, transmissibility and vaccine efficacy have been extensively characterized [5], much less is known about the underlying evolutionary mechanisms that drive their dynamics.

Substantial research, both theoretical and empirical, has been focused on understanding other aspects of the evolutionary dynamics of infectious disease: the long-term coexistence of diverse strains [6–8], the process of evolutionary rescue facilitating spillover into a new host species [9, 10], and the evolution of drug resistance during therapy [11–13]. However, less attention has been paid to the early-time dynamics of emerging pathogen variants during the epidemic phase of a disease. What factors contributed to the extremely rapid replacement of resident SARS-CoV-2 strains by each successive variant? Is the timing of variant detection and takeover primarily determined by the time of introduction or the stage of the epidemic in the population? When do we expect to see variants that primarily confer a transmission advantage vs those that primarily evade existing immune responses? When using models for scenario planning during epidemics, what scenarios for variant emergence should be considered? Are any aspects of variant emergence predictable?

Existing studies of early variant dynamics have tended to focus on quantifying the selective advantage of emerging variants compared to circulating strains. Many different models have been developed for this purpose [14–19], often producing different estimates. Existing estimation methods are also often sensitive to location-specific factors like population structure and levels of immunity that affect the speed of variant spread. For example, Earnest et. al [20] estimated that the SARS-CoV-2 Delta variant has a 63%–167% selective advantage over the Alpha variant (range of averages across US New England states), and Van Dorp et. al [16] estimated a 42%–125% selective advantage of the Omicron variant over Delta (range of medians across 40 countries). To add to this complexity, early dynamics of variants are subject to stochastic effects due to small population sizes right after emergence, which can obscure true selective advantages; stochasticity has been shown to accelerate early epidemic growth and substantially influence initial trajectories, complicating efforts to characterize emerging variants [21]. Moreover, identifying the nature of this advantage—whether the variant has selective advantage due to its immune evasive properties or due to a higher transmission rate or some combination of the two—is also important as it affects variant impact on mitigation measures and epidemic planning scenarios. However, different combinations of transmission advantage and immune evasion can result in the same overall selective advantage, making it impossible to identify the relative contributions of each solely using data on variant frequencies over time [22]. Identifying differences in variant emergence patterns depending upon their type could help in disentangling these contributions and improve estimation methods. Most importantly, methods that focus solely on quantifying the dynamics of observable variants cannot contribute to our understanding of the likelihood of variant emergence.

In this study we systematically evaluate the factors that determine the probability of variant invasion during outbreaks, the speed at which new variants spread and displace existing strains, and the overall impact of variant emergence on the epidemic trajectory. Using a simplified evo-epidemiological model, we examined how variant phenotype (increased transmissibility or immune evasion), source (local evolution vs importation), timing of introduction, distribution of population susceptibility, and the contact network structure (e.g. highly over-dispersed, clustered) interact to determine the fate of new variants. Inspired by SARS-CoV-2 variants of concern but designed to give general insight, we believe this approach is a necessary step towards developing better methods to interpret real-world data on variant frequencies and relate such data to underlying phenotypic effects. Moreover, we hope to provide intuition for when different types of variants are most likely to be detected during ongoing epidemics.

## Methods

### Model

**Figure 1:**
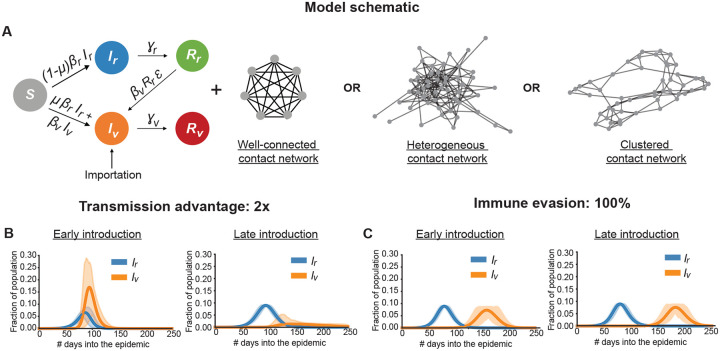
Model schematic and example infection dynamics. A) Schematic of the two strain SIR-type model with the different types of contact networks considered consisting of individuals susceptible to infection (*S*), infected by the resident (*I*_*r*_) or variant (*I*_*v*_) strain, and recovered from infection by the resident (*R*_*r*_) or variant (*R*_*v*_) strain. For strain i, *β*_*i*_ is the per contact transmission rate and *γ*_*i*_ is the rate of recovery. The variant can be produced via mutation (rate *μ*) of the resident strain or imported into the population from an outside source. Variants with immune evasive properties (0 < *ϵ* <= 1) can infect individuals recovered from the resident strain infection by a rate proportional to the strength of immune evasion *ϵ*. Example infection dynamics for a variant with B) transmission advantage and with C) immune evasive properties when introduced early or late in the epidemic. Results show the mean and 95% CI over 100 iterations.

We developed a simplified stochastic SIR-type model of a resident and a variant strain to study the dynamics of variant invasion and spread (Figure 1). Individuals in the population are classified as susceptible to infection (*S*); infected with the resident (*I*_*r*_) or the variant (*I*_*v*_) strain; or recovered from infection with the resident (*R*_*r*_) or the variant strain (*R*_*v*_). The resident and variant strain have different per contact rates of transmission to susceptible individuals (*β*_*r*_ and *β*_*v*_, respectively). We define the transmission advantage as *σ* = *β*_*v/*_*β*_*r*_. The infectious period can follow an arbitrary distribution with average duration 1_*/*_*γ*_*i*_ for strain *i*, which here we assume to be equal for both strains. Recovered individuals are assumed to be immune to re-infection in the absence of immune escape variants. We model immune evasion by defining an escape parameter *ϵ* (0 ≤ *ϵ* ≤ 1) which determines the efficiency at which the variant can infect individuals with immunity to the resident strain. When *ϵ* = 0, the variant has no immune evasive properties and can not infect individuals with immunity to the resident strain (i.e., in *R*_*r*_). When *ϵ* = 1, the variant achieves 100% immune evasion and can infect these previously-infected individuals as if they were fully susceptible. In the Supplement, we consider model extensions, such as allowing immunity to re-infection with the same strain to wane on same the time-scale as the initial epidemic peak and variant emergence.

We consider two modes of variant introduction: importation, where at pre-specified times in the simulation a variant infection is introduced into the population (e.g., from an outbreak in another population), and local evolution, where each infection with the resident strain can lead to transmission of a variant infection (i.e., due to within-host evolution) with a fixed probability *μ*.

We simulate infection spreading stochastically over a fixed contact network. For our main analysis, we consider a well-connected population where individuals can potentially infect anyone else. Later, we compare these results with those from more realistic contact patterns that take into account the effects of heterogeneity in the number of contacts and clustering of immunity (Figures 1A).

Similar to most models of infectious disease spread, the fitness of each strain can be encapsulated by the basic reproductive number, *R*_0_, which is defined as the average number of new infections generated by one infected individual introduced in a population of fully susceptible individuals, and its real-time analogue *R*_*t*_. For our model parameters, *R*_0_ for the two strains and *R*_*t*_ for the variant at the time of its introduction *t*_*int*_ obtained from their respective single strain models is given by,

(1) $$R_{0}^{r}=\frac{\beta (n-1)}{\gamma },\;R_{0}^{v}=\frac{\sigma \beta (n-1)}{\gamma },\;R_{t}^{v}\left(t_{\text{int}}\right)=\frac{\sigma \beta (n-1)}{\gamma }\frac{1}{N}\left[S\left(t_{\text{int}}\right)+\epsilon R_{r}\left(t_{\text{int}}\right)\right]$$

where *N* and *n* are the total population size and the mean degree of the network respectively. As a baseline, we assume $R_{0}^{r}=1.5$ and that both the strains have the same gamma-distributed duration of the infectious period (7 ± 4 days). The per contact transmission rate *β* is then calculated via Equation 1 for given *n*, *σ* and *ϵ*. Example dynamics of this model are provided in the Figures 1, S1, and S2. These values were chosen to approximately match the infectious period of SARS-CoV-2 [23], but the qualitative results we obtain are unaffected by this choice of parameter values.

Our stochastic model is simulated as a discrete time stochastic process and is implemented in the Python package JAX Numpy which is optimized to run simulations on GPUs, allowing us to simulate the whole epidemic in a few minutes even for population sizes of a million. Our model and implementation code is open access and available on GitHub: https://github.com/anjalika-nande/variant-emergence-patterns.

### Simulation details

#### Calculating the invasion probability of the variant

We first seed a resident strain infection (*I*_*r*_(*t* = 0) = 20) into a fully susceptible population of size 10,000. There are two ways in which a variant can then be introduced into the simulation at pre-specified times: a susceptible individual acquires a variant infection from outside of the population (importation), or the variant evolves in an individual infected with the resident strain (local evolution). We assume variant importation for most of the results presented here, but also consider local evolution. The introduction method does not affect the subsequent variant invasion dynamics in well-mixed populations, but in more structured contact networks the clustering of infected individuals can result in different invasion probabilities for variants that emerge via local evolution vs via importation. We choose variant importation times based on the cumulative size of the resident strain epidemic at the time of introduction. This allows us to study the impact of population immunity on variant invasion. We consider a range of epidemic sizes, from 0.5% – 50% of the population infected by the resident strain at the time of variant introduction.

To calculate the invasion probability of the variant, we run multiple simulations (10, 000 replicates) of our stochastic model with the mutation probability set to zero and for each iteration introduce one individual infected by the variant at the desired stage of the simulation. The simulation is run until zero infected individuals of either type remain. The invasion probability is then calculated as the fraction of iterations where the variant avoided early stochastic extinction. Practically, we classify an invasion as successful if the final epidemic size of the variant reaches at least 100 infections, a threshold we found reliably distinguishes sustained spread from early stochastic extinction and is independent of overall population size. Note that our invasion probability is *not* analogous to a measure of fixation probability in traditional population genetics models—there is no real concept of fixation here as both disease strains eventually go extinct as susceptibility wanes and the epidemic runs its course. Thus we choose an arbitrary (but we believe biologically relevant) threshold to define invasion, and for example, our invasion probabilities for neutral variants will not correspond to long-term expectations based on genetic drift.

#### Calculating variant prevalence over time

To investigate the dynamics of invading variants, we track prevalence of the variant as a function of time since introduction for simulations in which the variant successfully invaded. These sets of simulations were run for a larger population size of 1 million to represent a typical metropolitan area. Note, this larger population size was not used for the invasion probability simulations since running 10,000 replicates would have been computationally too time consuming and would not have altered the invasion probability. Prevalence is defined as the fraction of infections at any given time that are due to the variant (e.g., *I*_*v/*_(*I*_*r*_ + *I*_*v*_)). The variant is said to have invaded if it cumulatively infects at least 100 individuals and prevalence is averaged only for these successful invasions. We analyze trajectories of 50 successful invasions for each set of parameter values. For each replicate of the simulation we calculate the time for the variant to reach 5%, 50%, and 95% prevalence after introduction. We call the time at which the variant reaches 5% the ‘detection’ time and the time taken for the variant to sweep from 5% – 95% prevalence the ‘takeover’ time.

#### Simulating variant evolution

To simulate local evolution of the variant, we assume that, each new infection caused by an individual infected with the resident strain has a probability *μ* of instead being a variant infection. This is an over-simplification as we are encapsulating all the potentially relevant processes underlying the creation of the variant—such as, evolution through step-wise mutations or evolution in chronically infected individuals—in a single parameter. But this assumption is valid for our purposes to investigate the population-level invasion dynamics of variants produced via within-host de novo evolution in infected individuals since it captures the effects of more infections leading to a higher chance of variant production, and a faster rate of evolution leading to an earlier introduction of the variant.

We define ‘low’, ‘intermediate’ and ‘high’ rates of evolution depending upon the number of resident strain infections needed on average before 1 variant is produced. A low rate corresponds to one variant infection produced, on average, after 1 million resident strain infections, an intermediate rate after 10^5^ infections, and a high rate after 10^4^ infections. This translates to *μ* equaling 1_*e*_^*−*6^, 1_*e*_^*−*5^ and 1_*e*_^*−*4^ for low to high respectively. For reference, in the United Kingdom, the Alpha variant of SARS-CoV-2 was detected after *~* 1.3 × 10^4^ cumulative confirmed cases of the ancestral strain per million individuals [24,25].

### Details of contact networks

#### Well-connected network

Following [62], we approximate a well-mixed population by randomly connecting each person in the population to 100 other individuals. Although an ideal “well-mixed” network would involve connecting every individual to everyone else, given the large population sizes that we use (up to 1 million), this would require an enormous amount of computational memory and compromise the efficiency of using sparse matrices to represent the networks. Considering that in reality individuals typically transmit infections to only a limited number of others before recovering, a uniformly random network with a high degree serves as a good approximation of a fully-connected network.

#### Network with heterogeneity in the number of contacts

To study how heterogeneity in the number of individual contacts affects variant invasion and spread, we construct a network where the number of contacts individuals have follows a negative binomial distribution. There is no further structure in the network and individuals are randomly connected to each other. For baseline parameters we choose the coefficient of variation *CV* = 0.7 and the mean network degree *μ*_*network*_ = 15 and back out the parameters of the negative binomial distribution using the following relationships,

(2) $$\mu_{NB}=\mu_{\text{network}}/\left(1+CV^{2}\right)$$

(3) $$\sigma_{NB}=CV\times \mu_{NB}.$$

We choose the mean network degree to be 15 to roughly reflect the average number of daily contacts individuals have reported in studies conducted in US [63] and in European [64] populations. The coefficient of variation was chosen to get sufficient spread in the number of contacts.

#### Network with clustering of contacts

To study the effect of clustering of infections and consequently of immunity on variant invasion and spread, we construct a network where there is a high chance that the contacts of an individual are also contacts of each other. One way of creating such clustered networks is by using the Watts-Strogatz small-world algorithm [32]. We use the NetworkX python package [66] implementation of this method via the connected_watts_strogatz_graph function to create the network structure. We assume that all individuals have the same number of contacts which is again set to 15 to roughly reflect the average number of contacts per person observed in the US. We choose 0.2 as the probability of rewiring as for a network with mean degree of 15 this produces a network with a clustering coefficient ~ 0.3 which is the order of magnitude of clustering observed among human social contacts [29, 67].

## Results

### Invasion probability and epidemic impact

**Figure 2:**
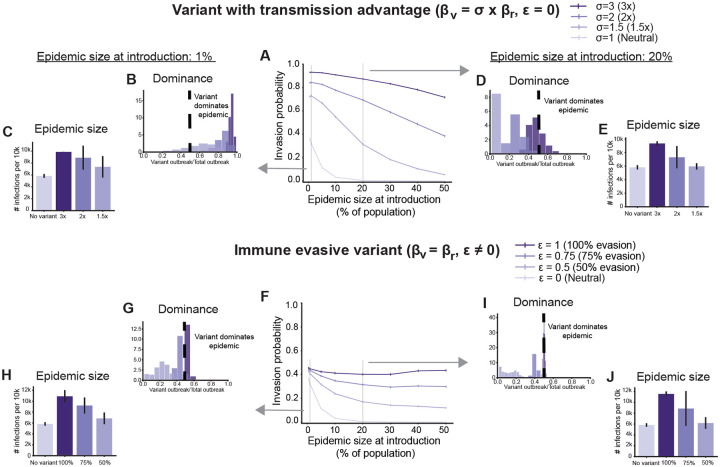
Effect of variant type and population immunity on variant invasion and epidemic impact. Results of stochastic simulations for variants with transmission advantage (top row) and variants with immune evasive properties (bottom row). A)&F) Fraction of simulations where the variant avoided early stochastic extinction as a function of the size of the resident strain epidemic at the time of introduction. The vertical lines are the standard error of proportion. Histograms showing the relative size of the variant outbreak compared to the total epidemic size, and the average total epidemic size conditional on non-extinction, for two introduction times: when the variant is introduced after 1% (B, C, G, H) or 20% (D, E, I, J) of the population has been infected by the resident strain. Vertical black lines on the epidemic size bars correspond to 2 standard deviations on either side of the mean. Darker colors correspond to variants with a higher advantage for both variant types. Results are for 10,000 replicates for each introduction time.

We first investigate how likely a new variant is to establish an outbreak depending upon its phenotype (enhanced transmissibility vs immune evasion) and what stage of the epidemic it first appears (Figures 1B, 2). We focus on two metrics of variant success: the fraction of simulations where the variant avoids early stochastic extinction, which is a proxy for the invasion probability, and the size of the variant outbreak relative to the whole outbreak (to gauge the overall impact of the variant on the epidemic). A neutral variant (identical to the resident strain) serves as a baseline. We note that the trends discussed here are unaffected by the specific choices of parameter values (see Suppl. Figures S3, S4, S5).

We find that for variants with a transmission advantage, the chance of invasion (versus extinction) increases with earlier introduction and with larger transmission advantage (Figure 2A). If introduced in the very early stages of an epidemic, even a neutral variant can have a significant chance of invasion purely on account of drift [26], and strains with even modest transmission advantages are highly likely to invade. For example, for the simulated scenarios, a neutral variant introduced after only 1% of the population had been previously infected had a 32% chance of invasion, compared to 73% for a strain with 1.5x transmission advantage and 92% with 3x transmission advantage. After early invasion, variants with a transmission advantage are likely to dominate the epidemic, increasing peak incidence and cumulative infections (Fig. 1B, 2B,C). If transmission advantage variants appear later in the epidemic, they are only likely to invade if they have large fitness advantages. For example, if they appear when 20% of the population has already been infected, the invasion probability drops to 0.39% for neutral variants, 30% for 1.5x advantage and only slightly—to 90%—for 3x transmission advantage. However, even if the chance of invasion is high, variants introduced later in the epidemic often fail to dominate the outbreak (Figure 2D). Even a highly transmissible variant (3x advantage) introduced after 20% prior infection dominates in only *~* 32% of simulations.

Population immunity at the time of introduction plays a big role in these trends. Although the relative fitness advantage of a variant compared to the resident strain does not depend on the introduction time, the absolute fitness at the time of introduction for a strain with only a transmission advantage is lower in more immune populations (Figure S6A). As a result, even if the transmission advantage of the variant is high, if introduced after a significant proportion of the population has already been infected, it can have only a limited impact. A consequence of this dynamic is that the overall impact of a successful variant on the epidemic—as measured by the increase in the total cumulative infections by the time the epidemic ends (also known as final attack size)—depends mainly on the variant fitness advantage and less so on the introduction time (Figures 2C,E). While variants introduced earlier tend to replace infections with the resident strain, the excess infections are similar and determined mainly by the difference in *R*_0_ between the strains.

For immune escape variants, we find that the chance of invasion increases with earlier introductions and higher levels of immune evasion (Figure 2F). As the epidemic progresses, unless immune escape is complete, immune escape variants have a lower invasion probability (higher probability of extinction) because there are fewer opportunities for secondary infections when prior immunity is higher (Figure S7). Note, however, that in the early epidemic stages when there is little immunity from prior infection, these variants are essentially neutral (Figure S6B) compared to the resident strain—they have the same baseline *R*_0_ and are competing for the exact same susceptible pool—and so the invasion probability is close to that of a neutral variant irrespective of the degree of immune escape. In agreement with previous work [27,28], we find that although the invasion probability can be substantial, immune escape variants rarely dominate the epidemic (Figures 2G,I). For example, a 50% immune evasive variant introduced after the resident strain has infected 1% of the population accounts for only 22% of total infections on average. This lack of dominance, however, does not imply a small overall epidemic, as depending upon the degree of immune evasion, the size of the variant outbreak can be close to that of the resident strain even if it does not exceed it. As a result, the final epidemic size can still be large and even exceed the population size for highly immune evasive variants due to reinfections (Figures 2H,J). While successfully invading variants with a transmission advantage tend to displace the resident strain, especially if they appear early in the epidemic or have large fitness advantages, immune escape strains cause less interference with the resident strain and instead owe their success to reinfections.

### Rate of increase in variant prevalence and consequences for detection

**Figure 3:**
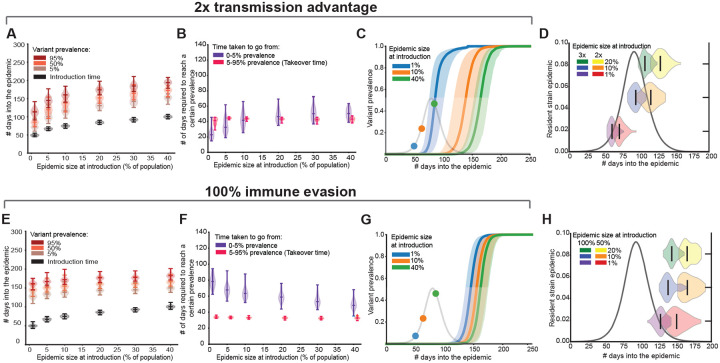
Rate of increase in variant prevalence as a function of the epidemic size at the time of introduction. Example results for a variant with transmission advantage (top row) and for an immune evasive variant (bottom row) when the variant escapes stochastic extinction. A)&E) Time when the variant was introduced (black violins) and reached 5%, 50% and 95% prevalence as a function of the size of the resident strain epidemic at the time of introduction. The violins are marked with the median, min and max values. B)&F) Number of days required for the variant to go from introduction to 5% and 5%–95% prevalence as a function of the size of the resident strain epidemic at the time of introduction. C)&G) Variant prevalence over time when introduced at different levels of population immunity. Solid line is the median and the shaded region corresponds to the 5–95 percentile range. The resident strain epidemic in the absence of the variant is overlaid in light grey along with the introduction times for reference. D)&H) Violin plots of the time at which the variant reaches 5% prevalence in reference to the resident strain epidemic (black curves) for different introduction times. Results are for 50 iterations for each introduction time in a population of size 1 million.

Next, for variants that successfully invade, we investigated the dynamics of their spread by tracking the fraction of all current infections caused by the variant over time (Figure 3). These variant frequency curves are commonly used to quantify variant fitness advantage from pathogen sequence data [14–17]. We find that variants with a transmission advantage increase in prevalence faster if they are introduced early in an epidemic compared to later (Figure 3A–C). This difference is primarily due to faster growth immediately after introduction. For example, a variant twice as transmissible as the resident strain reaches 5% prevalence in an average of 24 days if introduced when 1% of the population was already infected, but takes 42 days if not introduced until 10% prior infection (purple violins, Figure 3B). In contrast, the time taken to go from 5%–95% prevalence—which we define as the variant ‘takeover time’—is fairly consistent across introduction times (pink violins, Figure 3B). For immune escape variants, we find the opposite trend: the earlier they are introduced, the *longer* they take to reach 5% prevalence (Figures 3E–G). Immune evasive variants tend to linger at a low prevalence for long periods of time until a critical level of immunity has built up in the population due to infections with the resident strain epidemic, at which point the variant can quickly take over (Figure 3G). Note that these dynamics are conditional on non-extinction. Although these variants do not actually take over until much later in the epidemic, essentially washing out the effect of their introduction time, their higher probability of invasion in the early epidemic stages still makes it more advantageous for them to arise very early and spread neutrally with respect to the resident strain until they have enough of an advantage to really start out-competing it.

These trends can be explained by considering the two major regimes in variant invasion dynamics: a stochastic regime immediately after introduction, and a deterministic regime once the variant has escaped stochastic extinction. During the stochastic phase, which coincides roughly with the time from introduction until reaching 5% prevalence, the rate of increase in variant prevalence is influenced by both absolute and relative fitness of the variant. For variants with a transmission advantage, relative fitness is constant over time (Figure S6). Higher absolute fitness drives the observation that variants introduced earlier have a higher probability of invasion and rise in prevalence more rapidly. In contrast, for immune evasive variants, absolute fitness decreases over the course of the epidemic, leading to small decreases in invasion probability, but it is compensated for by increasing relative fitness, leading to faster establishment for later introduction times. Variant dynamics during the 5–95% prevalence sweep (‘takeover’) fall under the deterministic regime, where relative fitness primarily governs the dynamics. Since the relative fitness of variants with a transmission advantage is always constant, the takeover time is unaffected by introduction time. For immune escape variants, although relative fitness increases over time, variants introduced at different times tend to reach 5% prevalence at similar stages in the resident strain epidemic, resulting in similar relative advantages during the takeover phase and thus comparable takeover times.

These differences in variant dynamics have important real-world implications for when different types of variants are likely to be detected during an epidemic. If we approximate ‘detection time’ as the time taken to reach 5% prevalence, our result suggests that variants with a transmission advantage can be detected during any phase of the resident strain epidemic, depending on the timing of introduction and strength of their advantage, but that immune evasive variants, irrespective of their degree of immune evasion or introduction times, will most likely be detected only in the later stages when there is already a significant amount of population immunity (Figures 3D,H, and Supplementary Discussion and Figure S8). These trends are robust to the choice of parameter values (Figures S5, S9, S10)

**Figure 4:**
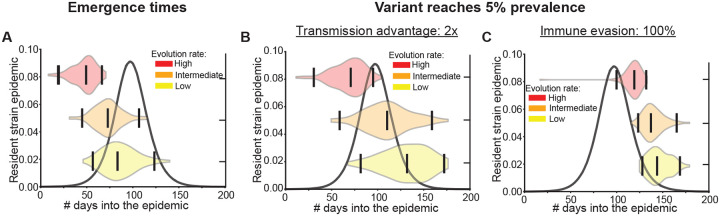
Variant emergence and detection times for different rates of evolution. Time at which variants A) emerge and B)-C) reach 5% prevalence during the resident strain epidemic when the rate of evolution is high (red), intermediate (orange), and low (yellow). The violin plots are marked with the median and the middle 95% quantile range. The resident strain epidemic in the absence of the variant is provided as a reference (black curve). Results are for 100 iterations and population size of a million. Low (10^*−*6^), medium (10^*−*5^), and high (10^*−*4^) evolutionary rates correspond to the emergence of a new variant, on average, after the number of infections equal to the entire population size, 10%, or 1% of the population, respectively. See Methods for more details.

So far we have modeled variant introduction as a single importation of the variant from an external source. We also considered what happens when variants are generated from current infections with the resident strain with some transition probability, meant to approximate the process of within-host evolution. We find that the trends observed for the timing of variant appearance still hold (Figure 4). For example, even when the rate of variant production is high, a variant that completely escapes immunity is typically detected around 25 days after the epidemic peak, with detection occurring even later for partially immune evasive variants (Figure S11).

### Effect of contact heterogeneity and clustering on variant dynamics

So far we’ve considered a well-connected population where an infected individual can infect anyone else. This assumption allowed us to isolate the effects of variant type and population immunity on a variant’s ability to invade and spread. However, in reality, human contacts are heterogeneous, they tend to be clustered, and these patterns are known to affect disease transmission in a complicated way [29–34]. To investigate the role played by such realistic human contact patterns on variant invasion dynamics, we repeat the previous analysis on two types of structured populations: a contact network where there is a high degree of heterogeneity in the number of individual contacts, and one where each individual has the same number of contacts but they are clustered, that is, there is a high chance that the contacts of an individual are also contacts of each other (see Methods for more details). This choice allows us to separately study the role played by contact heterogeneity and clustering of immunity on variant invasion dynamics.

**Figure 5:**
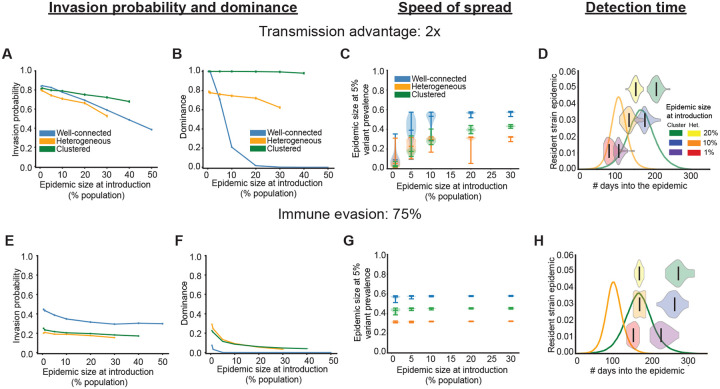
Effect of contact heterogeneity and clustering on variant invasion and spread. Example results for variants with a transmission advantage (top row) and with immune evasive properties (bottom row) when the transmission network is well-connected (blue), heterogeneous (orange) and clustered (green). A)&E) Fraction of simulations when the variant infected more than 1% of the population, and B)&F) fraction of invading runs when the variant dominated the epidemic as a function of the size of the resident strain epidemic at introduction. Vertical marks are the standard error in proportion and results are for ~ 10^4^ iterations. C)&G) Epidemic size when the variant reaches 5% prevalence versus the epidemic size when it was introduced. D)&H) Violin plots of the time at which the variant reaches 5% prevalence in reference to the resident strain epidemic for the heterogeneous (orange curve) and clustered networks (green curve). Solid middle line is the median and results are for ~ 50 iterations. See Methods for more details.

We first investigated the effects of heterogeneity and clustering on the variant invasion probability (Figure 5, Figure S12). Across all network types, we observe the same trends as for the well-connected case—irrespective of variant type, invasion becomes less likely later in the resident strain epidemic as the availability of susceptible individuals decreases. However, compared to the well-connected scenario, variants generally find it harder to invade in heterogeneous and clustered networks; with the exception being that for variants with a transmission advantage, clustering of immunity can lower or increase the chance of invasion depending upon the population immunity at the time of emergence and the strength of the transmission advantage.

A key difference between structured and well-mixed populations is that, in structured populations, the chance of invasion depends on where in the network the variant emerges; for example, a variant is less likely to invade when it is introduced in an individual with fewer contacts as compared to a well-connected one. Consequently, heterogeneity in contact patterns generally lowers the average invasion probability compared to the well-connected case. This also means that the source of the variant—whether imported or locally evolved—matters. When we assume the variant evolves locally, instead of being imported, we find similar qualitative trends (Figures S13, S14). However, for variants with a transmission advantage, the invasion probability is lower under local emergence, since they are more likely to arise in parts of the network with fewer susceptible individuals.

Next, we examine the dynamics of variants that successfully invade. Compared to the well-connected case, heterogeneity and clustering can help variants dominate the epidemic (Figures 5B,F) by helping them spread through the population at a faster rate (Figures 5C,G). Variants that successfully invade usually emerge in the more connected and susceptible parts of the network which allows them to out-compete the resident strain more easily than in the well-connected case. One way to quantify this is by comparing the variant’s rise in prevalence relative to the overall epidemic progression. For example, when a variant twice as transmissible as the resident strain is introduced into a population with 10% prior infection, it reaches 5% prevalence when median cumulative infections are ~ 50% in the well-connected case, but only ~30% in structured networks. As a result, the variant dominates the epidemic 100% and 80% of the time in clustered and heterogeneous networks respectively, versus just 20% in the well-connected case. However, although variants increase in prevalence faster, for each network type, our earlier finding that compared to variants with a transmission advantage (Figures 5D and S15A,B), immune evasive variants are more likely to be detected when there are higher levels of population immunity, still holds (Figures 5H and S15C,D).

## Discussion

Continual pathogen evolution is one of the main barriers to infectious disease control, with adaptations commonly leading to escape from immune responses. The COVID-19 pandemic is a recent example of this, where despite vaccines being developed at record-breaking speeds, the successive emergence and dominance of novel viral variants evading immunity has led to a continual burden of disease [35]. Recurring annual outbreaks of seasonal influenza are sustained by similar dynamics, where antigenic evolution occurs both via accumulation of de novo mutations and through genomic reassortment in animal reservoirs [36,37]. Similarly, sustained transmission and sporadic outbreaks of norovirus, particularly the dominant GII.4 strain, is made possible by recurrent emergence of new variants [38,39]. Although more difficult to measure, viral variants with increased transmissibility have also contributed to emergent or recurrent burden of many infections (e.g. for SARS-CoV-2 [40,41], influenza [42], West Nile virus [43], Ebola [44]).

Despite innovations in our ability to track pathogen genomic evolution in real-time [45] and measure strain-specific immunity using a high-throughput methods [46–50], the COVID-19 pandemic accentuated the many open questions about the evolutionary dynamics of novel viral variants. What factors contributed to how quickly successive variants emerged from 2021 onwards? Are there differences in the expected likelihood, timing, and dynamics of emergence of variants with a transmission advantage versus immune escape properties? Does superspreading help or hinder variant emergence? Do we expect variant emergence to be synchronized across geographic regions with different infection histories? Understanding all these factors is essential to design epidemic control strategies that can appropriately respond to the ever changing pathogen landscape.

In this study, we use a stochastic epidemic model to examine the invasion dynamics of variants with either transmission advantage or immune evasive properties, in the presence of varying levels of population immunity. We find that, irrespective of the underlying transmission network structure, variants with a transmission advantage or partial immune evasion are more likely to invade and cause large outbreaks when they first appear early in an epidemic when the number of susceptible hosts is high, as they are in direct competition for hosts with existing strains. In contrast, highly immune evasive variants always have access to a largely susceptible population, and their chance of invasion and outbreak size is insensitive to timing of first appearance. While the invasion of immune escape variants can significantly increase epidemic size, these strains are less likely to dominant and outcompete resident strains unless they completely evade existing immunity.

The dynamics of successfully invading variants can be roughly divided into two phases: an initial stochastic phase, where the variant avoids extinction, and a subsequent deterministic one. Our results suggest that the invasion dynamics of the two variant types differ predominantly in the stochastic phase. Variants with a transmission advantage benefit from low levels of population immunity and escape this phase faster the earlier they are introduced in the epidemic, with the rate increasing for increasing levels of advantage. In contrast, successfully invading immune-escape variants increase in prevalence at a much slower rate in the early epidemic stages irrespective of the strength of immune evasion. These variants persist at low prevalence for extended periods, spreading nearly neutrally relative to existing strains until they gain a sufficient relative advantage due to increasing levels of population immunity to existing strains. This suggests that immune evasive variants may evade detection until the later stages of an epidemic. Indeed this is one possible explanation for why the SARS-CoV-2 Omicron variant was detected much later than when phylodynamic analysis suggests it first evolved [51]. Similar patterns have been observed for norovirus variants [52]. As the stochastic phase of invasion typically precedes detection, improving identifiability of the nature of variant fitness advantages will likely require integrating diverse data sources that inform this early phase, such as phylodynamic estimates of introduction times or human mobility-informed models of viral importation [53,54]. Ultimately, all such approaches would benefit from earlier and more sensitive detection of variant introductions, which could be facilitated by enhanced surveillance systems such as wastewater monitoring [55,56] or targeted sampling at points of entry (e.g., airports [57,58]).

To date, most characterization of variant invasion dynamics has focused on measuring the relative instantaneous growth rate (or effective time-varying reproduction number, *R*_*t*_) of the emerging strain based on case incidence data once the variant has reached a detectable frequency, often using popular software packages (e.g., EpiEstim R package [15,69]). While such methods will certainly be limited by not informing the probability of invasion or the early stochastic phase of emergence, we hypothesized they are also limited by not explicitly including inter-strain competition for susceptible individuals when estimating variant advantage. Employing EpiEstim on simulation results for transmission-advantage variants (Suppl. Analysis), we found that this approach generally leads to an overestimation of the variant advantage, but occasionally led to underestimation. For situations where the variant was weakly advantageous (e.g. *~* 1.5x transmissibility) and was introduced into an established epidemic (> 10% infected) the error in the estimated advantage was only a few percent (Fig.S16). However, the error was more pronounced for higher fitness variants, earlier introduction times, and increased levels of mixing in the population, with errors up to 25%.

Two prior studies examined the evolutionary epidemiology of emerging variants inspired by SARS-CoV-2 [27,28]. A key difference of our work is that by using a stochastic model, we are able to examine the invasion probability, timing, and early dynamics, which prior work could not. Examining only the more deterministic phase of variant spread, Bushman *et al* [27] also found that immune escape variants rarely dominate the epidemic, though transmission advantage mutants often do. Reynes *et al* [28] also found the important role of timing of emergence on the subsequent impact of immune escape variants, and found effects suggestive of delayed emergence in some cases.

Our study has several limitations. We haven’t yet considered variants that have *both* a transmission advantage and immune evasive properties, since our focus was on disentangle the differences between the two types of selective advantages. At least in the case of SARS-CoV-2, many variants were suspected to have both advantages, and prior models have included this [27], though it’s also possible that an emerging strain with an improved value of one trait may pay a fitness cost in the other trait. Further work is needed to understand the dynamics of such variants. We don’t include the effects of waning immunity in our model, but our preliminary exploration (and a related deterministic model [28]) suggests that the qualitative trends of invasion observed here are unchanged, and we still find that immune evasive variants are most likely to be detected after the epidemic peaks (see Suppl. Analysis and Figure S17). However, the long-term interaction between waning immunity and immune evasiveness in endemic settings is complicated [59], and left for future work. We also haven’t considered how interventions such as vaccines may affect variants, particularly immune evasive strains (see, e.g. [27, 28]), and especially if there are different population subgroups with different vaccine coverage. While this study focuses on the population-level emergence and selection of variants, these strains must first be generated and selected within individual hosts. Within-host selection is affected by factors not considered here, such as the duration of infection, the relationship between transmissibility and pathogen load, and the dynamics of innate and adaptive immune responses [60, 61]. Finally, we have yet to formally compare the predictions of our models to the extensive spatio-temporal data on SARS-CoV-2 variants, or related data for other pathogens.

In conclusion, our results highlight the epidemiological and evolutionary factors influencing the invasion potential of new pathogen variants during an ongoing outbreak. They shed light on dynamics that drove (and continue to drive) the global burden of COVID-19 and other rapidly-evolving infections, and generate hypotheses that can be tested with pathogen genomic surveillance data. Incorporating the dynamics of variant emergence processes into models, alongside improved surveillance, will enable more accurate risk assessments and, in turn, better inform public health policies.

## Supplementary Material

1

## Data Availability

No data was acquired for this study. All code used to generate the results presented here is open source and available on Github: https://github.com/anjalika-nande/variant-emergence-patterns.

## References

[1] Carabelli AM, Peacock TP, Thorne LG, Harvey WT, Hughes J, de Silva TI, SARS-CoV-2 variant biology: immune escape, transmission and fitness. Nature Reviews Microbiology. 2023 Mar;21:162–77. 36653446 10.1038/s41579-022-00841-7 PMC9847462

[2] Wilton T, Bujaki E, Klapsa D, Majumdar M, Zambon M, Fritzsche M, Rapid Increase of SARS-CoV-2 Variant B.1.1.7 Detected in Sewage Samples from England between October 2020 and January 2021. mSystems. 2021;6(3):e00353–21. 34128696 10.1128/mSystems.00353-21 PMC8269227

[3] Mlcochova P, Kemp SA, Dhar MS, Papa G, Meng B, Ferreira IATM, SARS-CoV-2 B.1.617.2 Delta variant replication and immune evasion. Nature. 2021 Nov;599(7883):114–9. 34488225 10.1038/s41586-021-03944-y PMC8566220

[4] Chatterjee S, Bhattacharya M, Nag S, Dhama K, Chakraborty C. A Detailed Overview of SARS-CoV-2 Omicron: Its Sub-Variants, Mutations and Pathophysiology, Clinical Characteristics, Immunological Landscape, Immune Escape, and Therapies. Viruses. 2023 Jan;15(1):167. 36680207 10.3390/v15010167 PMC9866114

[5] Markov PV, Ghafari M, Beer M, Lythgoe K, Simmonds P, Stilianakis NI, The evolution of SARS-CoV-2. Nature Reviews Microbiology. 2023 Jun;21:361–79. 37020110 10.1038/s41579-023-00878-2

[6] Gog JR, Grenfell BT. Dynamics and selection of many-strain pathogens. Proceedings of the National Academy of Sciences. 2002 Dec;99(26):17209–14. Available from: 10.1073/pnas.252512799. PMC139294 12481034

[7] Kucharski AJ, Andreasen V, Gog JR. Capturing the dynamics of pathogens with many strains. Journal of Mathematical Biology. 2015 Mar;72(1–2):1–24. Available from: 10.1007/s00285-015-0873-4. 25800537 PMC4698306

[8] Makau DN, Lycett S, Michalska-Smith M, Paploski IAD, Cheeran MCJ, Craft ME, Ecological and evolutionary dynamics of multi-strain RNA viruses. Nature Ecology & Evolution. 2022 Sep;6(10):1414–22. Available from: 10.1038/s41559-022-01860-6. 36138206

[9] Nuismer SL, Basinski AJ, Schreiner C, Whitlock A, Remien CH. Reservoir population ecology, viral evolution and the risk of emerging infectious disease. Proceedings of the Royal Society B: Biological Sciences. 2022;289(1982):20221080. 10.1098/rspb.2022.1080 PMC9470272 36100013

[10] Shaw CL, Kennedy DA. Developing an empirical model for spillover and emergence: Orsay virus host range in Caenorhabditis. Proceedings of the Royal Society B: Biological Sciences. 2022 Sep;289(1983):20221165. 10.1098/rspb.2022.1165 PMC9489279 36126684

[11] Alexander H, Bonhoeffer S. Pre-existence and emergence of drug resistance in a generalized model of intra-host viral dynamics. Epidemics. 2012;4(4):187–202. 23351371 10.1016/j.epidem.2012.10.001

[12] Pennings PS. HIV Drug Resistance: Problems and Perspectives. Infectious Disease Reports. 2013 Jun;5:e5. 10.4081/idr.2013.s1.e5 PMC3892620 24470969

[13] Lehtinen S, Blanquart F, Croucher NJ, Turner P, Lipsitch M, Fraser C. Evolution of antibiotic resistance is linked to any genetic mechanism affecting bacterial duration of carriage. Proceedings of the National Academy of Sciences of the United States of America. 2017 Jan;114(5):1075–80. 28096340 10.1073/pnas.1617849114 PMC5293062

[14] van Dorp CH, Goldberg EE, Hengartner N, Ke R, Romero-Severson EO. Estimating the strength of selection for new SARS-CoV-2 variants. Nature Communications. 2021 Dec;12(1). Available from: 10.1038/s41467-021-27369-3. PMC8671537 34907182

[15] Bhatia S, Wardle J, Nash RK, Nouvellet P, Cori A. Extending EpiEstim to estimate the transmission advantage of pathogen variants in real-time: SARS-CoV-2 as a case-study. Epidemics. 2023 Sep;44:100692. Available from: 10.1016/j.epidem.2023.100692. 37399634 PMC10284428

[16] van Dorp C, Goldberg E, Ke R, Hengartner N, Romero-Severson E. Global estimates of the fitness advantage of SARS-CoV-2 variant Omicron. Virus Evolution. 2022 10;8(2):veac089. Available from: 10.1093/ve/veac089. PMC9615435 36325031

[17] Davies NG, Abbott S, Barnard RC, Jarvis CI, Kucharski AJ, Munday JD, Estimated transmissibility and impact of SARS-CoV-2 lineage B.1.1.7 in England. Science. 2021 Apr;372(6538). Available from: 10.1126/science.abg3055. PMC8128288 33658326

[18] Suchard MA, Lemey P, Baele G, Ayres DL, Drummond AJ, Rambaut A. Bayesian phylogenetic and phylodynamic data integration using BEAST 1.10. Virus Evolution. 2018 Jan;4(1). 10.1093/ve/vey016 PMC6007674 29942656

[19] Bouckaert R, Vaughan TG, Barido-Sottani J, Duchêne S, Fourment M, Gavryushkina A, BEAST 2.5: An advanced software platform for Bayesian evolutionary analysis. PLOS Computational Biology. 2019 Apr;15(4). 10.1371/journal.pcbi.1006650 PMC6472827 30958812

[20] Earnest R, Uddin R, Matluk N, Renzette N, Turbett SE, Siddle KJ, Comparative Transmissibility of SARS-CoV-2 Variants Delta and Alpha in New England, USA. Cell Reports Medicine. 2022 Apr;3(4):100583. 35480627 10.1016/j.xcrm.2022.100583 PMC8913280

[21] Czuppon P, Schertzer E, Blanquart F, Débarre F. The stochastic dynamics of early epidemics: probability of establishment, initial growth rate, and infection cluster size at first detection. Journal of The Royal Society Interface. 2021;18(184):20210575. 34784776 10.1098/rsif.2021.0575 PMC8596012

[22] Viana R, Moyo S, Amoako DG, Tegally H, Scheepers C, Althaus CL, Rapid epidemic expansion of the SARS-CoV-2 Omicron variant in southern Africa. Nature. 2022 Mar;603(7902):679–86. 35042229 10.1038/s41586-022-04411-y PMC8942855

[23] Nande A, Sheen J, Walters EL, Klein B, Chinazzi M, Gheorghe AH, The effect of eviction moratoria on the transmission of SARS-CoV-2. Nature Communications. 2021 Apr;12(1). Available from: 10.1038/s41467-021-22521-5. PMC8050248 33859196

[24] Hodcroft EB. “CoVariants: SARS-CoV-2 Mutations and Variants of Interest.”; 2021. Available from: https://covariants.org/.

[25] Mathieu E, Ritchie H, Rodés-Guirao L, Appel C, Gavrilov D, Giattino C, COVID-19 Pandemic. Our World in Data. 2020. Https://ourworldindata.org/coronavirus.

[26] Kinnunen M, Dechesne A, Proctor C, Hammes F, Johnson D, Quintela-Baluja M, A conceptual framework for invasion in microbial communities. The ISME Journal. 2016 May;10(12):2773–9. Available from: 10.1038/ismej.2016.75. 27137125 PMC5148196

[27] Bushman M, Kahn R, Taylor BP, Lipsitch M, Hanage WP. Population impact of SARS-CoV-2 variants with enhanced transmissibility and/or partial immune escape. Cell. 2021 Dec;184(26):6229–42.e18. Available from: 10.1016/j.cell.2021.11.026. 34910927 PMC8603072

[28] Reyné B, Djidjou-Demasse R, Sofonea MT, Alizon S. Mutant emergence timing and population immunisation status impact epidemiological dynamics. Journal of Theoretical Biology. 2025 Jul;608. 10.1016/j.jtbi.2025.112140 40348170

[29] Leventhal GE, Hill AL, Nowak MA, Bonhoeffer S. Evolution and emergence of infectious diseases in theoretical and real-world networks. Nature Communications. 2015 Jan;6(1). Available from: 10.1038/ncomms7101. PMC4335509 25592476

[30] Lloyd-Smith JO, Schreiber SJ, Kopp PE, Getz WM. Superspreading and the effect of individual variation on disease emergence. Nature. 2005 Nov;438(7066):355–9. Available from: 10.1038/nature04153. 16292310 PMC7094981

[31] Read JM, Keeling MJ. Disease evolution on networks: the role of contact structure. Proceedings of the Royal Society of London Series B: Biological Sciences. 2003 Apr;270(1516):699–708. Available from: 10.1098/rspb.2002.2305. PMC1691304 12713743

[32] Watts DJ, Strogatz SH. Collective dynamics of ‘small-world’ networks. Nature. 1998 Jun;393(6684):440–2. Available from: 10.1038/30918. 9623998

[33] Moreno Y, Pastor-Satorras R, Vespignani A. Epidemic outbreaks in complex heterogeneous networks. The European Physical Journal B. 2002 Apr;26(4):521–9. Available from: 10.1140/epjb/e20020122.

[34] May RM, Lloyd AL. Infection dynamics on scale-free networks. Physical Review E. 2001 Nov;64(6). Available from: 10.1103/physreve.64.066112. 11736241

[35] Barfar E, Raei B, Daneshi S, Bagher Barahouei F, Hushmandi K. The burden of COVID-19 based on disability-adjusted life years: a systematic review of available evidence. Frontiers in Public Health. 2025 Feb;13. Publisher: Frontiers. 10.3389/fpubh.2025.1401726 PMC11891345 40066002

[36] Petrova VN, Russell CA. The evolution of seasonal influenza viruses. Nature Reviews Microbiology. 2018 Jan;16(1):47–60. Available from: http://www.nature.com/articles/nrmicro.2017.118. 29081496 10.1038/nrmicro.2017.118

[37] Han AX, de Jong SPJ, Russell CA. Co-evolution of immunity and seasonal influenza viruses. Nature Reviews Microbiology. 2023 Dec;21(12):805–17. Available from: https://www.nature.com/articles/s41579-023-00945-8. 37532870 10.1038/s41579-023-00945-8

[38] Parra GI. Emergence of norovirus strains: A tale of two genes. Virus Evolution. 2019;5(2). Available from: 10.1093/ve/vez048. PMC6875644 32161666

[39] Tohma K, Lepore CJ, Gao Y, Ford-Siltz LA, Parra GI. Population Genomics of GII.4 Noroviruses Reveal Complex Diversification and New Antigenic Sites Involved in the Emergence of Pandemic Strains. mBio. 2019 Sep;10(5):10.1128/mbio.02202-19. Available from: https://journals.asm.org/doi/10.1128/mbio.02202-19. PMC6759766 31551337

[40] Korber B, Fischer WM, Gnanakaran S, Yoon H, Theiler J, Abfalterer W, Tracking Changes in SARS-CoV-2 Spike: Evidence that D614G Increases Infectivity of the COVID-19 Virus. Cell. 2020 Aug;182(4):812–27.e19. Available from: http://www.sciencedirect.com/science/article/pii/S0092867420308205. 32697968 10.1016/j.cell.2020.06.043 PMC7332439

[41] Davies NG, Abbott S, Barnard RC, Jarvis CI, Kucharski AJ, Munday JD, Estimated transmissibility and impact of SARS-CoV-2 lineage B.1.1.7 in England. Science. 2021 Apr;372(6538). Available from: https://science.sciencemag.org/content/372/6538/eabg3055. 10.1126/science.abg3055 PMC8128288 33658326

[42] Dorigatti I, Cauchemez S, Ferguson NM. Increased Transmissibility Explains the Third Wave of Infection by the 2009 H1N1 Pandemic Virus in England. Proceedings of the National Academy of Sciences. 2013 Aug;110(33):13422–7. 10.1073/pnas.1303117110 PMC3746905 23882078

[43] Bialosuknia SM, Dupuis AP II, Zink SD, Koetzner CA, Maffei JG, Owen JC, Adaptive Evolution of West Nile Virus Facilitated Increased Transmissibility and Prevalence in New York State. Emerging Microbes & Infections. 2022 Dec;11(1):988–99. 35317702 10.1080/22221751.2022.2056521 PMC8982463

[44] Diehl WE, Lin AE, Grubaugh ND, Carvalho LM, Kim K, Kyawe PP, Ebola Virus Glycoprotein with Increased Infectivity Dominated the 2013–2016 Epidemic. Cell. 2016 Nov;167(4):1088–98.e6. 27814506 10.1016/j.cell.2016.10.014 PMC5115602

[45] Hadfield J, Megill C, Bell SM, Huddleston J, Potter B, Callender C, Nextstrain: Real-Time Tracking of Pathogen Evolution. Bioinformatics. 2018 Dec;34(23):4121–3. 29790939 10.1093/bioinformatics/bty407 PMC6247931

[46] Fonville JM, Wilks SH, James SL, Fox A, Ventresca M, Aban M, Antibody Landscapes after Influenza Virus Infection or Vaccination. Science. 2014 Nov;346(6212):996–1000. 25414313 10.1126/science.1256427 PMC4246172

[47] Xu GJ, Kula T, Xu Q, Li MZ, Vernon SD, Ndung’u T, Comprehensive Serological Profiling of Human Populations Using a Synthetic Human Virome. Science. 2015 Jun;348(6239):aaa0698. 10.1126/science.aaa0698 PMC4844011 26045439

[48] Shrock EL, Shrock CL, Elledge SJ. VirScan: High-throughput Profiling of Antiviral Antibody Epitopes. Bio-protocol. 2022 Jul;12(13). 10.21769/BioProtoc.4464 PMC9303818 35937932

[49] Kendra JA, Tohma K, Ford-Siltz LA, Lepore CJ, Parra GI. Antigenic Cartography Reveals Complexities of Genetic Determinants That Lead to Antigenic Differences among Pandemic GII.4 Noroviruses. Proceedings of the National Academy of Sciences. 2021 Mar;118(11):e2015874118. 10.1073/pnas.2015874118 PMC7980451 33836574

[50] Wang W, Lusvarghi S, Subramanian R, Epsi NJ, Wang R, Goguet E, Antigenic Cartography of Well-Characterized Human Sera Shows SARS-CoV-2 Neutralization Differences Based on Infection and Vaccination History. Cell Host & Microbe. 2022 Dec;30(12):1745–58.e7. 36356586 10.1016/j.chom.2022.10.012 PMC9584854

[51] Dor G, Wilkinson E, Martin DP, Moir M, Tshiabuila D, Kekana D, Tracing the spatial origins and spread of SARS-CoV-2 Omicron lineages in South Africa. Nature Communications. 2025 May;16(1):4937. 10.1038/s41467-025-60081-0 PMC12120024 40436832

[52] Ruis C, Lindesmith LC, Mallory ML, Brewer-Jensen PD, Bryant JM, Costantini V, Preadaptation of Pandemic GII.4 Noroviruses in Unsampled Virus Reservoirs Years before Emergence. Virus Evolution. 2020 Jul;6(2):veaa067. 10.1093/ve/veaa067 PMC7751145 33381305

[53] Lemey P, Rambaut A, Bedford T, Faria N, Bielejec F, Baele G, Unifying Viral Genetics and Human Transportation Data to Predict the Global Transmission Dynamics of Human Influenza H3N2. PLOS Pathogens. 2014 Feb;10(2). 10.1371/journal.ppat.1003932 PMC3930559 24586153

[54] Chen Z, Tsui JLH, Gutierrez B, Busch Moreno S, du Plessis L, Deng X, COVID-19 pandemic interventions reshaped the global dispersal of seasonal influenza viruses. Science. 2024 Nov;386(6722). 10.1126/science.adq3003 PMC11760156 39509510

[55] Jahn K, Dreifuss D, Topolsky I, Kull A, Ganesanandamoorthy P, Fernandez-Cassi X, Early detection and surveillance of SARS-CoV-2 genomic variants in wastewater using COJAC. Nature Microbiology. 2022 Aug;7(8):1151–60. 10.1038/s41564-022-01185-x PMC9352586 35851854

[56] Sapoval N, Liu Y, Lou EG, Hopkins L, Ensor KB, Schneider R, Enabling accurate and early detection of recently emerged SARS-CoV-2 variants of concern in wastewater. Nature Communications. 2023 May;14(1):2834. 10.1038/s41467-023-38184-3 PMC10191095 37198181

[57] Overton AK, Knapp JJ, Lawal OU, Gibson R, Fedynak AA, Adebiyi AI, Genomic surveillance of Canadian airport wastewater samples allows early detection of emerging SARS-CoV-2 lineages. Scientific Reports. 2024 Nov;14(1):26534. 39489759 10.1038/s41598-024-76925-6 PMC11532424

[58] St-Onge G, Davis JT, Hébert-Dufresne L, Allard A, Urbinati A, Scarpino SV, Pandemic monitoring with global aircraft-based wastewater surveillance networks. Nature Medicine. 2025;31:788–96. 10.1038/s41591-025-03501-4 PMC11922747 39939526

[59] Otto SP, MacPherson A, Colijn C. Endemic does not mean constant as SARS-CoV-2 continues to evolve. Evolution. 2024 Jun;78(6). 10.1093/evolut/qpae041 38459852

[60] Morris DH, Petrova VN, Rossine FW, Parker E, Grenfell BT, Neher RA, Asynchrony between Virus Diversity and Antibody Selection Limits Influenza Virus Evolution. eLife. 2020 Nov;9:e62105. 33174838 10.7554/eLife.62105 PMC7748417

[61] Otto SP, Day T, Arino J, Colijn C, Dushoff J, Li M, The Origins and Potential Future of SARS-CoV-2 Variants of Concern in the Evolving COVID-19 Pandemic. Current Biology. 2021 Jul;31(14):R918–29. 34314723 10.1016/j.cub.2021.06.049 PMC8220957

