# Supplementary material for: Understanding patterns of variant emergence and spread in an ongoing epidemic
Source: medRxiv. 2026 Mar 30:2026.03.27.26349560. Preprint. [Version 1] doi: 10.64898/2026.03.27.26349560 (PMC13060446; doi:10.64898/2026.03.27.26349560)
Supplement: 1 [file NIHPP2026.03.27.26349560V1-supplement-1.pdf]

medRxiv preprint doi: https://doi.org/10.64898/2026.03.27.26349560; this version posted March 30, 2026. The copyright holder for this preprint (which was not certified by peer review) is the author/funder, who has granted medRxiv a license to display the preprint in perpetuity.
It is made available under a CC-BY 4.0 International license .

Supplementary Figures

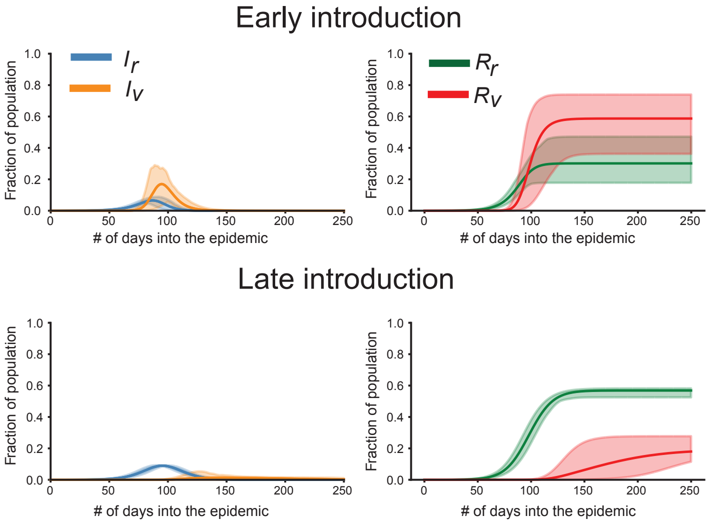

Figure S1: Example dynamics of the two strain model with a transmission advantage variant. Time-course of the number of infected (left) and recovered for the two strains (right) when the variant is introduced early (top row) and late (bottom) in the resident strain epidemic. Early (late) introduction corresponds to the variant introduced when the resident strain has infected 1% (10%) of the population. Results are for resident strain $R_0^r = 1.5$ and a variant that is twice as transmissible $R_0^v = 3$. The solid line and shaded region corresponding to the mean and the 5th-95th percentile calculated from 50 iterations of the simulation.

medRxiv preprint doi: https://doi.org/10.64898/2026.03.27.26349560; this version posted March 30, 2026. The copyright holder for this preprint (which was not certified by peer review) is the author/funder, who has granted medRxiv a license to display the preprint in perpetuity.
It is made available under a CC-BY 4.0 International license .

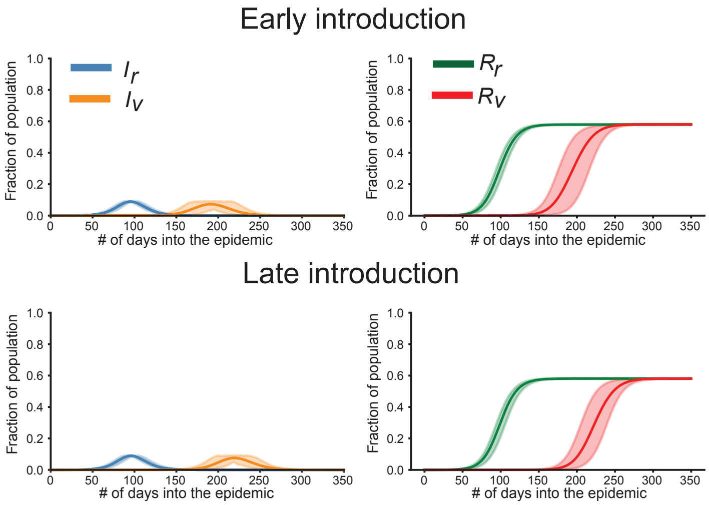

Figure S2: Example dynamics of the two strain model with a immune evasive variant. Time-course of the number of infected (left) and recovered for the two strains (right) when the variant is introduced early (top row) and late (bottom) in the resident strain epidemic. Early (late) introduction corresponds to the variant introduced when the resident strain has infected 1% (20%) of the population. Results are for resident strain $R_0^r = 1.5$ and a fully immune evasion variant ($\epsilon = 1$) variant with no transmission advantage $R_0^v = 1.5$. The solid line and shaded region corresponding to the mean and the 5th-95th percentile calculated from 50 iterations of the simulation.

medRxiv preprint doi: https://doi.org/10.64898/2026.03.27.26349560; this version posted March 30, 2026. The copyright holder for this preprint (which was not certified by peer review) is the author/funder, who has granted medRxiv a license to display the preprint in perpetuity.
It is made available under a CC-BY 4.0 International license .

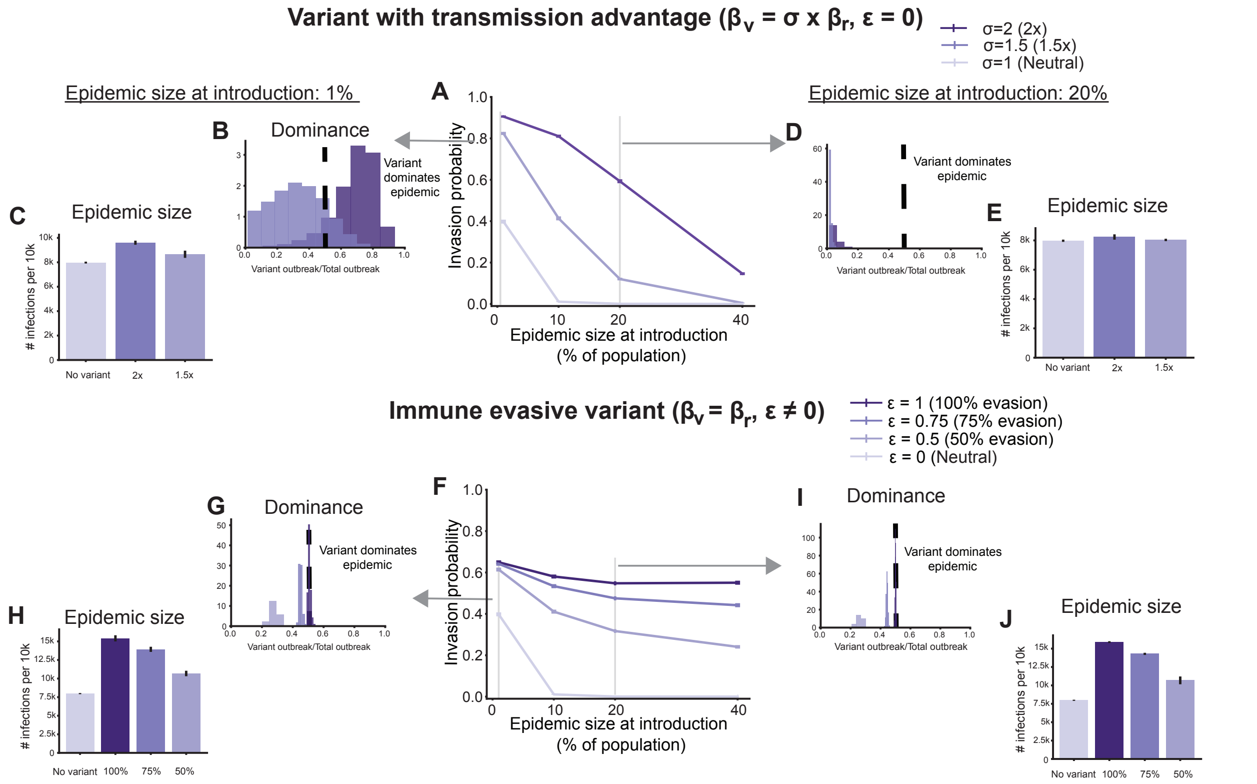

Figure S3: Effect of variant type and population immunity on variant invasion and epidemic impact when resident strain $R_0 = 2$. Results of stochastic simulations for variants with transmission advantage (top row) and variants with immune evasive properties (bottom row). A)&F) Fraction of simulations where the variant infected more than 1% of the population as a function of the size of the resident strain epidemic at the time of introduction. The vertical lines are the standard error of proportion. Histograms of the relative size of the variant outbreak compared to the total epidemic size and the average total epidemic size for two introduction times: variant introduced when B)&C), G)&H) 1% and D)&E), I)&J) 20% (bottom) of the population has been infected by the resident strain. Darker colors correspond to variants with a higher advantage for both variant types. Results are for 10,000 iterations for each introduction time.

medRxiv preprint doi: https://doi.org/10.64898/2026.03.27.26349560; this version posted March 30, 2026. The copyright holder for this preprint (which was not certified by peer review) is the author/funder, who has granted medRxiv a license to display the preprint in perpetuity.
It is made available under a CC-BY 4.0 International license .

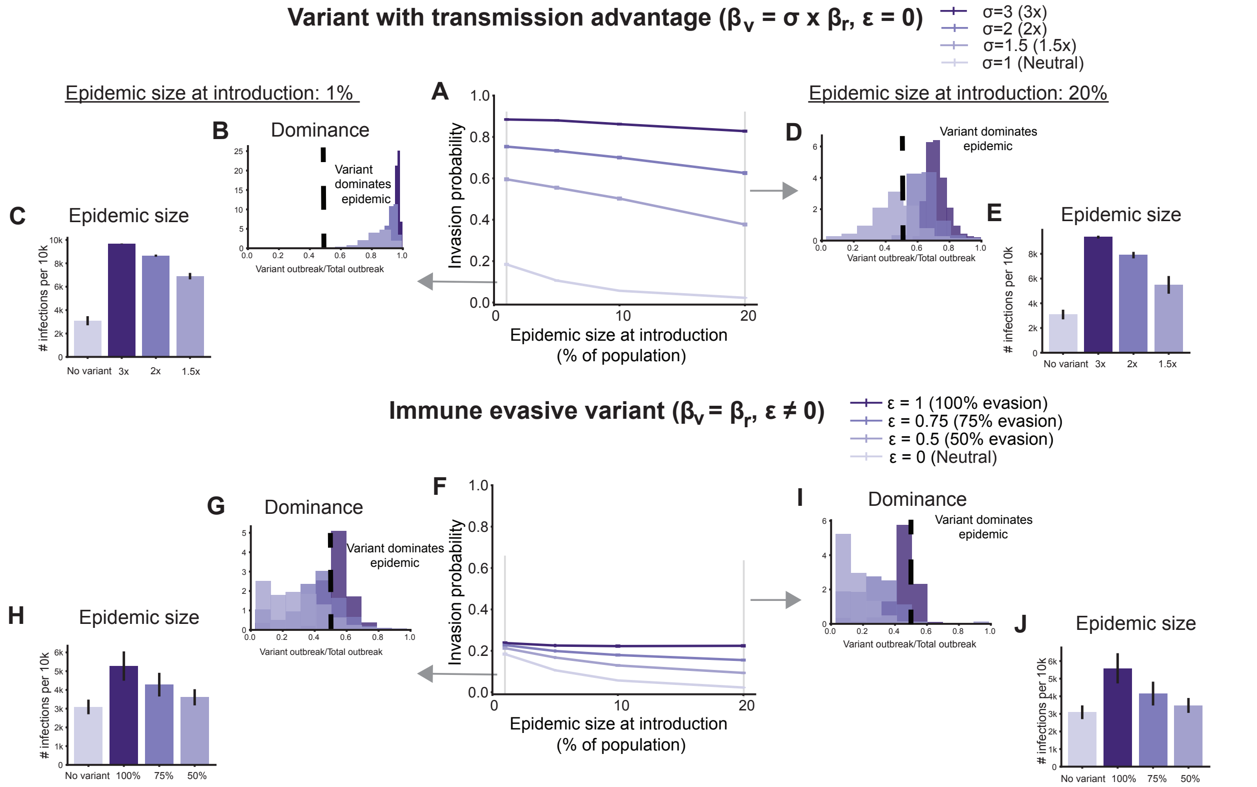

Figure S4: Effect of variant type and population immunity on variant invasion and epidemic impact when resident strain $R_0 = 1.2$. Results of stochastic simulations for variants with transmission advantage (top row) and variants with immune evasive properties (bottom row). A)&F) Fraction of simulations where the variant infected more than 1% of the population as a function of the size of the resident strain epidemic at the time of introduction. The vertical lines are the standard error of proportion. Histograms of the relative size of the variant outbreak compared to the total epidemic size and the average total epidemic size for two introduction times: variant introduced when B)&C), G)&H) 1% and D)&E), I)&J) 20% (bottom) of the population has been infected by the resident strain. Darker colors correspond to variants with a higher advantage for both variant types. Results are for 10,000 iterations for each introduction time.

medRxiv preprint doi: https://doi.org/10.64898/2026.03.27.26349560; this version posted March 30, 2026. The copyright holder for this preprint (which was not certified by peer review) is the author/funder, who has granted medRxiv a license to display the preprint in perpetuity.
It is made available under a CC-BY 4.0 International license .

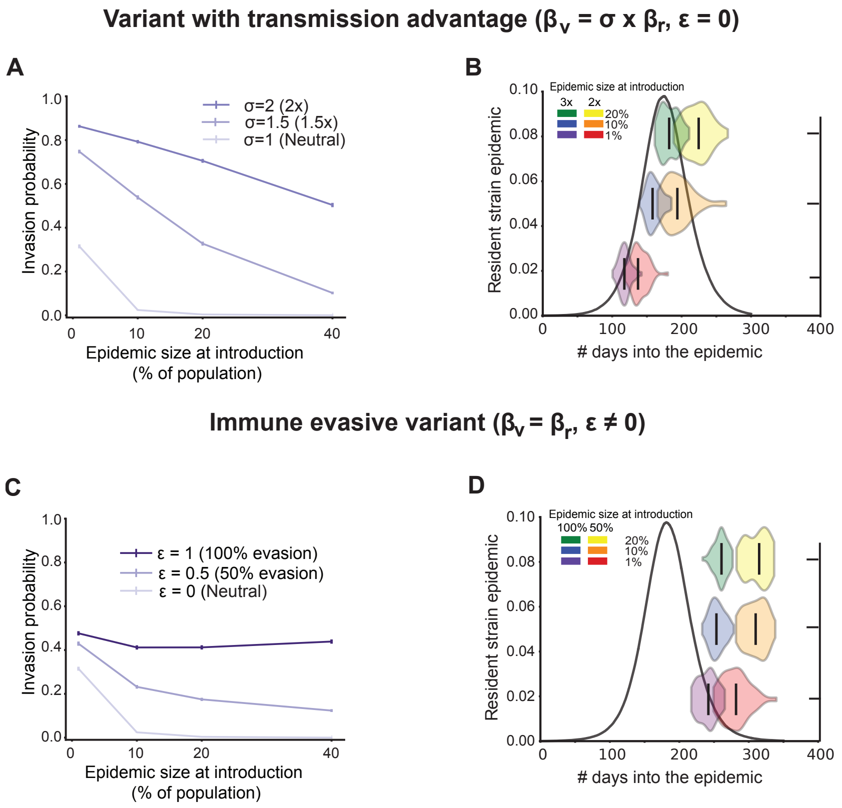

Figure S5: Invasion probability and detection times for variant under a longer duration of baseline infectiousness. Example results for a variant with transmission advantage (top row) and for an immune evasive variant (bottom row) with $R_0 = 1.5$ and gamma-distributed duration of infectiousness with mean 14 and standard deviation of 7 days. A)& C) Fraction of simulations where the variant infected more than 1% of the population as a function of the size of the resident strain epidemic at the time of introduction. Darker colors correspond to variants with a higher advantage for both variant types. Results are for 10,000 iterations for each introduction time with the vertical lines denoting the standard error of proportion. B)&D) Violin plots of the time at which the variant reaches 5% prevalence in reference to the resident strain epidemic (black curves) for different introduction times. Results are for ~ 50 iterations for each introduction time.

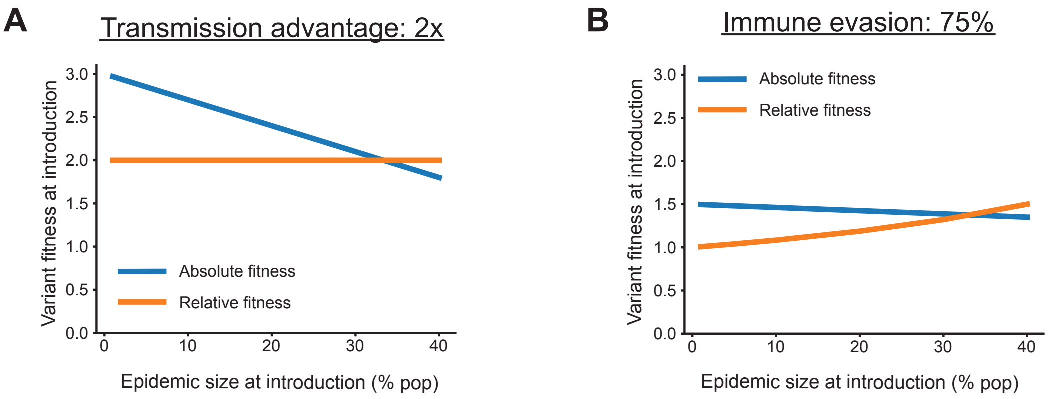

Figure S6: Example variant fitness at the time of introduction. The absolute and relative fitness of a variant as a function of the resident strain epidemic size at the time of its introduction into a resident strain epidemic when it is A) twice as transmissible as the resident strain and B) when it is 75% immune evasive. Resident strain $R_0 = 1.5$

medRxiv preprint doi: https://doi.org/10.64898/2026.03.27.26349560; this version posted March 30, 2026. The copyright holder for this preprint (which was not certified by peer review) is the author/funder, who has granted medRxiv a license to display the preprint in perpetuity.
It is made available under a CC-BY 4.0 International license .

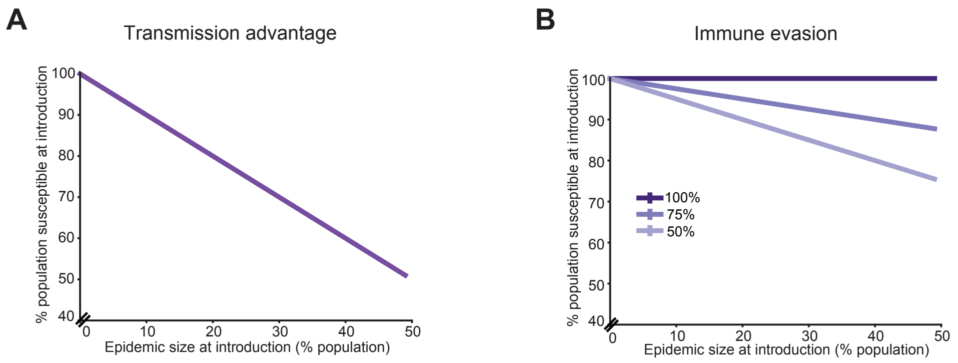

Figure S7: Amount of population susceptibility at the time of variant introduction. The percent of population that is susceptible to getting infected by the variant as a function of the resident strain epidemic size at the time of its introduction for A) a variant with transmission advantage and B) a variant with immune evasive properties. We provide a few levels of immune evasion as an example.

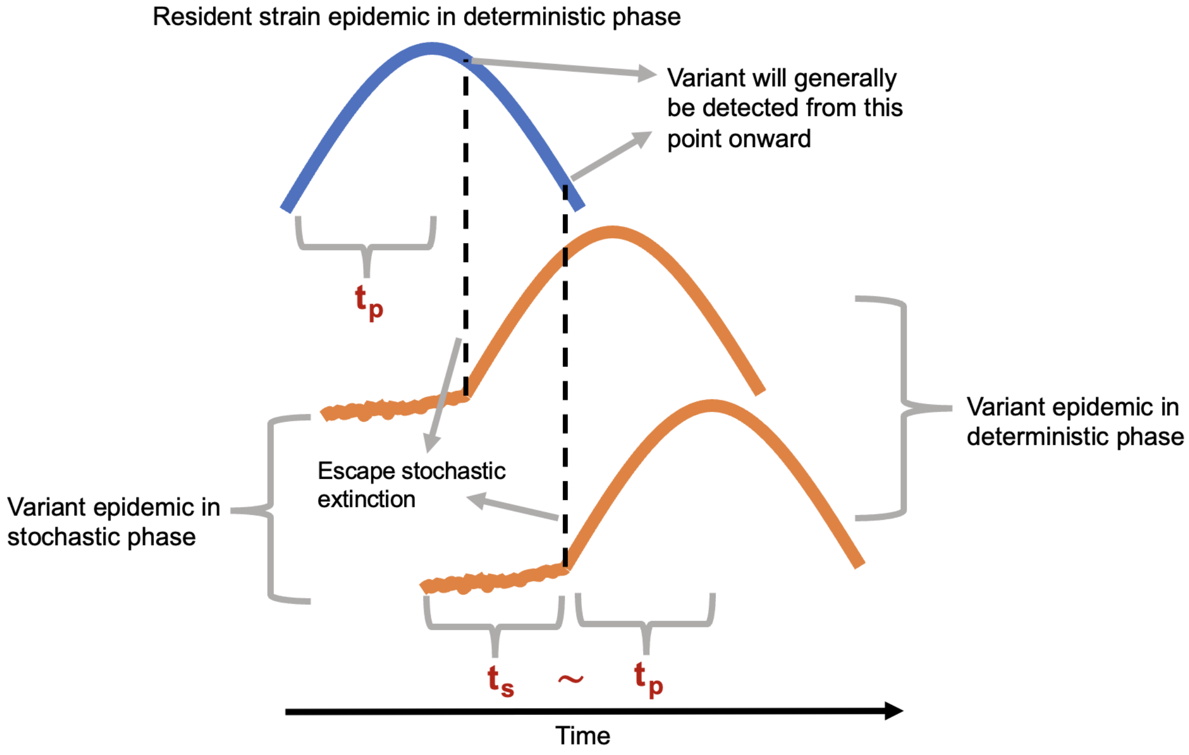

Figure S8: Schematic to provide intuition for time-scales in the variant invasion process. Blue curve is the schematic of an epidemic curve for the resident strain that is already in its deterministic phase of spread. Orange curves correspond to the variant that first undergoes a stochastic phase for time t_s after which it enters in the deterministic phase once it escapes extinction. t_p is the time taken from that point onward to the epidemic peak. The deterministic trajectories of the two strains are shown to be the the same as it is a schematic of a fully immune evasive variant with the same baseline R_0. Using the birth-death process approximation we find $t_s \sim t_p$.

medRxiv preprint doi: https://doi.org/10.64898/2026.03.27.26349560; this version posted March 30, 2026. The copyright holder for this preprint (which was not certified by peer review) is the author/funder, who has granted medRxiv a license to display the preprint in perpetuity.
It is made available under a CC-BY 4.0 International license .

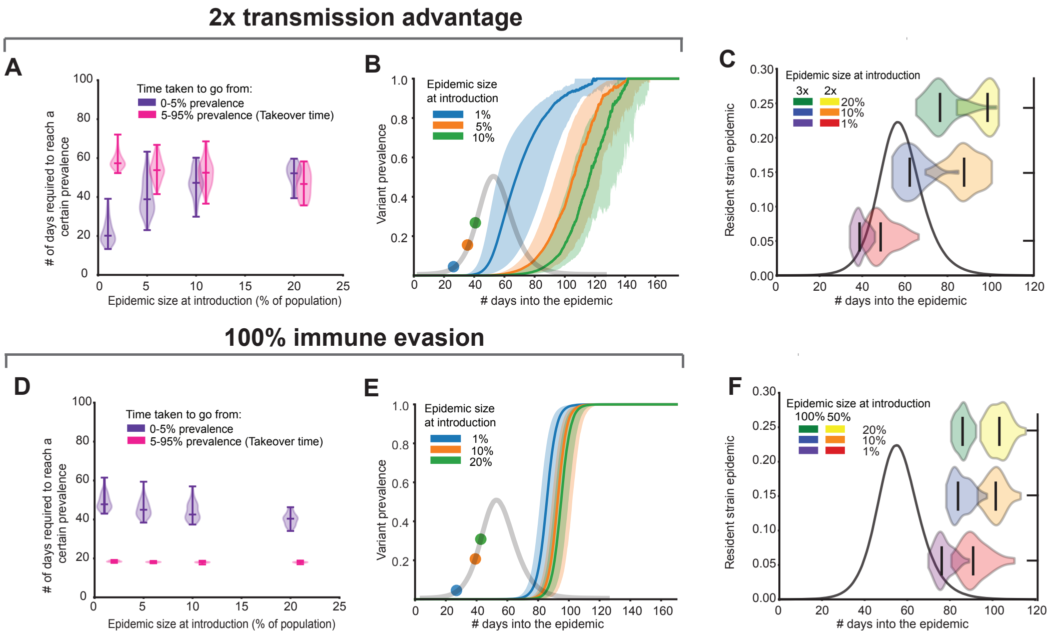

Figure S9: Rate of increase in variant prevalence as a function of the epidemic size at the time of introduction when resident strain $R_0 = 2$. Example results for a variant with transmission advantage (top row) and for an immune evasive variant (bottom row) when the variant escapes stochastic extinction. A)&D) Number of days required for the variant to go from introduction to 5% and $5\% - 95\%$ prevalence as a function of the size of the resident strain epidemic at the time of introduction. B)&E) Variant prevalence over time when introduced at different levels of population immunity. Solid line is the median and the shaded region corresponds to the 5-95 percentile range. The resident strain epidemic in the absence of the variant is overlaid in light grey and marked with the respective introduction times. C)&F) Violin plots of the time at which the variant reaches 5% prevalence in reference to the resident strain epidemic (black curves) for different introduction times. Results are for 50 iterations for each introduction time.

medRxiv preprint doi: https://doi.org/10.64898/2026.03.27.26349560; this version posted March 30, 2026. The copyright holder for this preprint (which was not certified by peer review) is the author/funder, who has granted medRxiv a license to display the preprint in perpetuity.
It is made available under a CC-BY 4.0 International license .

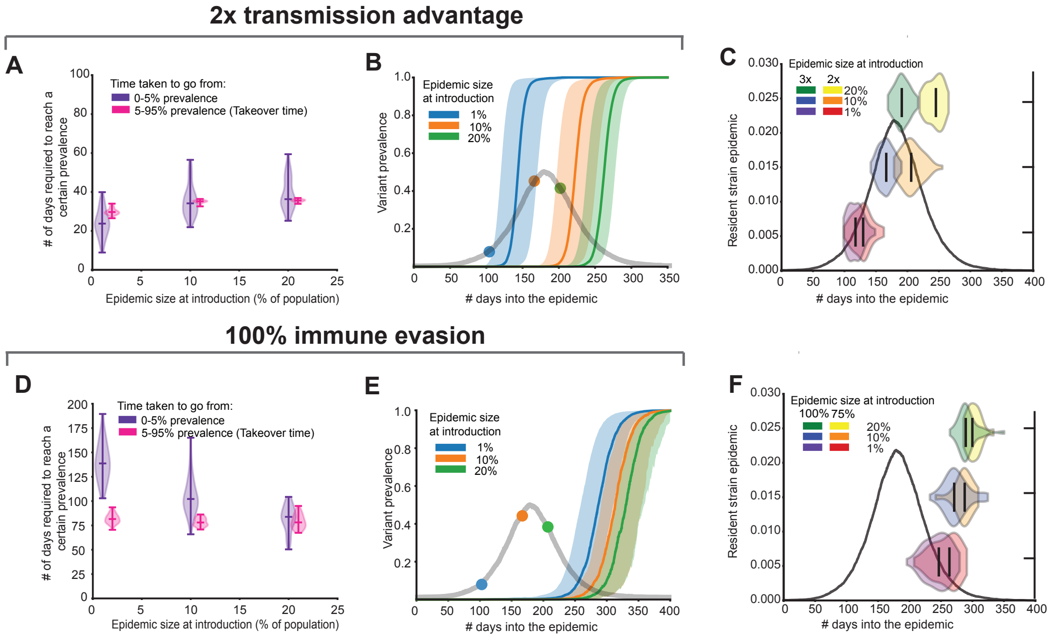

Figure S10: Rate of increase in variant prevalence as a function of the epidemic size at the time of introduction when resident strain $R_0 = 1.2$. Example results for a variant with transmission advantage (top row) and for an immune evasive variant (bottom row) when the variant escapes stochastic extinction. A)&D) Number of days required for the variant to go from introduction to 5% and $5\% - 95\%$ prevalence as a function of the size of the resident strain epidemic at the time of introduction. B)&E) Variant prevalence over time when introduced at different levels of population immunity. Solid line is the median and the shaded region corresponds to the 5-95 percentile range. The resident strain epidemic in the absence of the variant is overlaid in light grey and marked with the respective introduction times. C)&F) Violin plots of the time at which the variant reaches 5% prevalence in reference to the resident strain epidemic (black curves) for different introduction times. Results are for ~ 50 iterations for each introduction time.

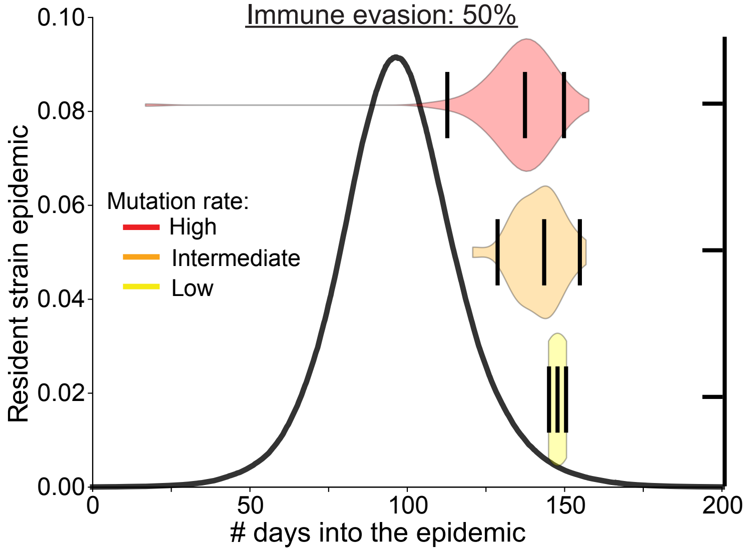

Figure S11: Variant detection times for different rates of evolution. Time at which a variant that is 50% immune evasive reaches 5% prevalence during the resident strain epidemic for different rates of evolution. The resident strain epidemic in the absence of the variant is provided as a reference (black curve). Results are for 100 iterations. The violin plots are marked with the median and the middle 95% quantile range.

medRxiv preprint doi: https://doi.org/10.64898/2026.03.27.26349560; this version posted March 30, 2026. The copyright holder for this preprint (which was not certified by peer review) is the author/funder, who has granted medRxiv a license to display the preprint in perpetuity.
It is made available under a CC-BY 4.0 International license .

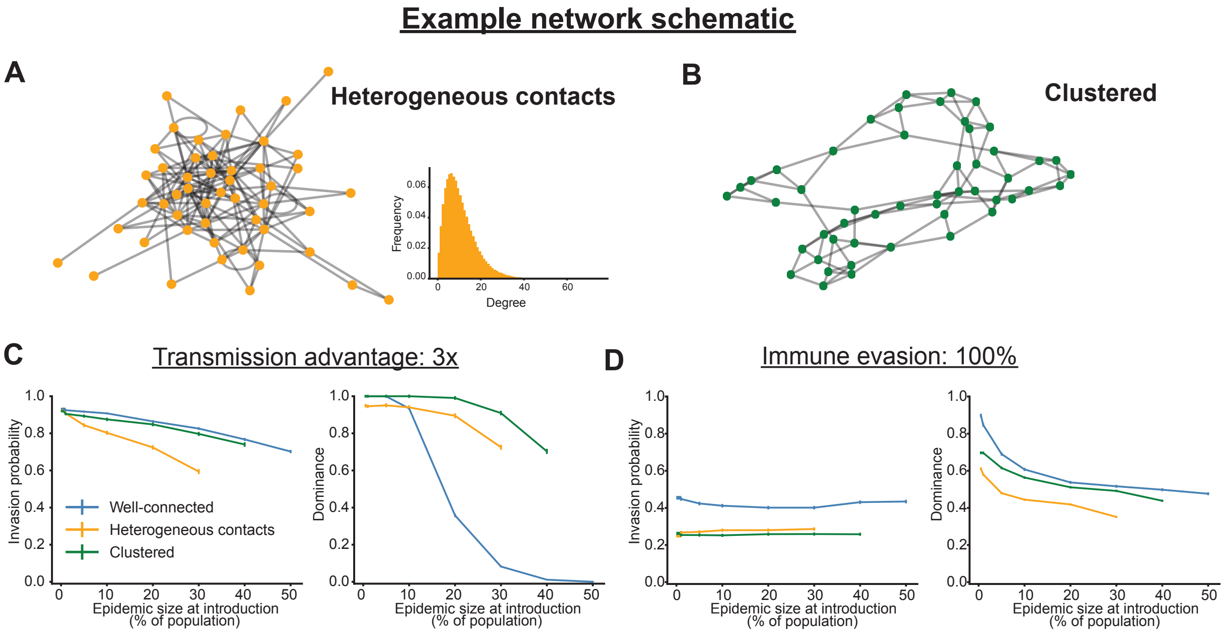

Figure S12: Schematic of the different types of networks considered and variant invasion and dominance results. A) Example of a network with heterogeneity in the number of contacts and the actual degree distribution used in this work. B) Example of a network with clustering of individuals where each individual as the same number of contacts. C)-D) Variant invasion probability and dominance for a C) transmission advantage and D) immune evasive variant for different underlying network structures. Invasion probability is defined as the fraction of simulations where the variant infected at least 1% of the population and dominance is the fraction of simulations where the variant dominated the epidemic. Results are for $5000 - 10,000$ iterations for each introduction time and error bars correspond to the standard error of proportion.

medRxiv preprint doi: https://doi.org/10.64898/2026.03.27.26349560; this version posted March 30, 2026. The copyright holder for this preprint (which was not certified by peer review) is the author/funder, who has granted medRxiv a license to display the preprint in perpetuity.
It is made available under a CC-BY 4.0 International license .

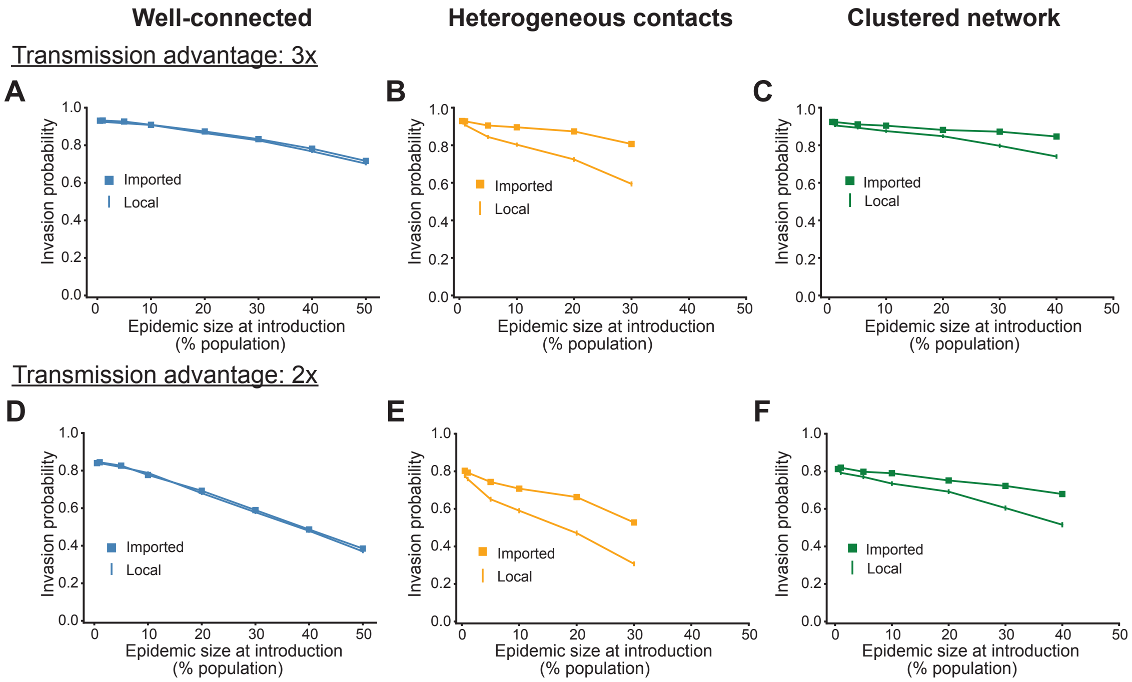

Figure S13: Invasion probability for two modes of variant introduction. Invasion probability of a variant as a function of the size of the epidemic when it is introduced for two example amounts of transmission advantage. Variant is either imported into (square) or evolves locally in the population (straight line) with different underlying structures. Results in subplots A)&D) are for a well-connected population, B)&E) for a population with heterogeneous number of contacts, and C)&F) clustered population. Invasion probability is defined as the fraction of simulations (n=10,000) where the variant infected at least 1% of the population. See SI Methods for network details.

medRxiv preprint doi: https://doi.org/10.64898/2026.03.27.26349560; this version posted March 30, 2026. The copyright holder for this preprint (which was not certified by peer review) is the author/funder, who has granted medRxiv a license to display the preprint in perpetuity.
It is made available under a CC-BY 4.0 International license .

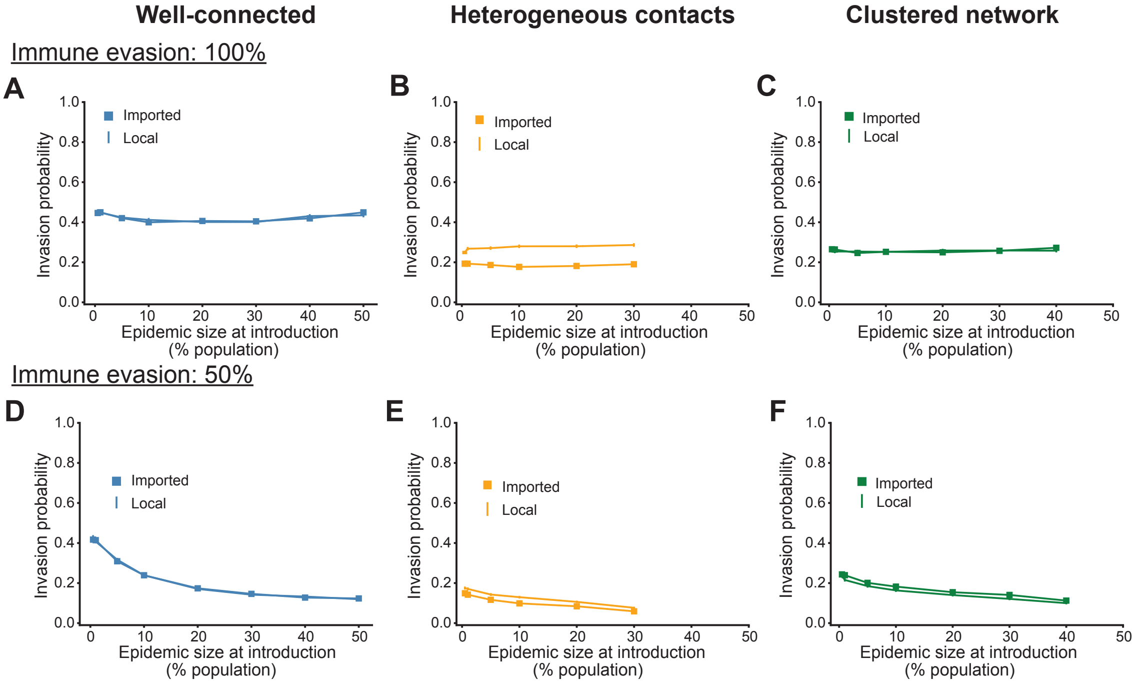

Figure S14: Invasion probability for two modes of variant introduction. Invasion probability of a variant as a function of the size of the epidemic when it is introduced for two example amounts of immune evasion. Variant is either imported into (square) or evolves locally in the population (straight line) with different underlying structures. Results in subplots A)&D) are for a well-connected population, B)&E) for a population with heterogeneous number of contacts, and C)&F) clustered population. Invasion probability is defined as the fraction of simulations (n=10,000) where the variant infected at least 1% of the population. See SI Methods for network details.

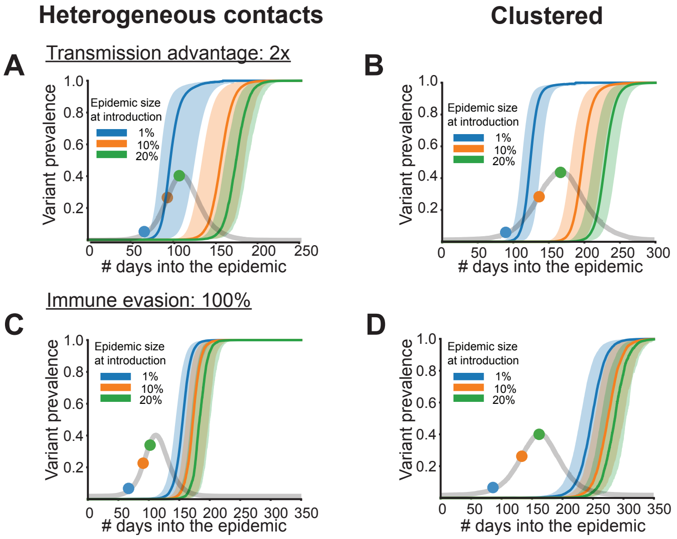

Figure S15: Dynamics of variant prevalence over time for the different underlying network structures. Variant prevalence as a function of time since the start of the resident strain epidemic for three example variant introduction times when the transmission network is either heterogeneous in the number of individual contacts (left) or is clustered (right). A)-B) Results are for a variant with a transmission advantage and C)-D) for an immune evasive variant. The resident strain epidemic in the absence of the variant is overlaid in grey along with the introduction times for reference. Results are for the baseline resident strain $R_0^r = 1.5$ and for 50 iterations for each introduction time in a population of size 1 million. The solid line and shaded region corresponds to the median and the 5-95 percentile range respectively.

medRxiv preprint doi: https://doi.org/10.64898/2026.03.27.26349560; this version posted March 30, 2026. The copyright holder for this preprint (which was not certified by peer review) is the author/funder, who has granted medRxiv a license to display the preprint in perpetuity.
It is made available under a CC-BY 4.0 International license .

Example results for a variant with 2x transmission advantage (well-connected)

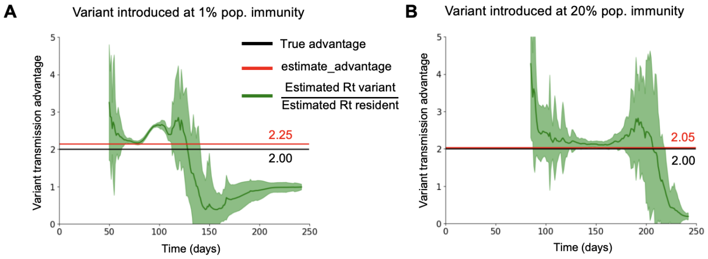

C **% Over/under estimation of transmission advantage (well-mixed)**

Transmission advantage	Population immunity at introduction		
	1%	10%	20%
1.5x	4.67	4.00	-3.33
2x	12.50	11.50	2.50
3x	25.33	15.00	14.33

General trend → Increasing advantage / Increasing overestimation ↓

D **% Over/under estimation of transmission advantage (heterogeneous network)**

Transmission advantage	Population immunity at introduction		
	1%	10%	20%
1.5x	4.00	4.00	4.00
2x	9.50	5.00	3.50
3x	22.67	12.33	11.33

E **% Over/under estimation of transmission advantage (clustered network)**

Transmission advantage	Population immunity at introduction		
	1%	10%	20%
1.5x	0.67	-1.33	-2.67
2x	3.50	-2.50	-3.00
3x	9.33	6.67	0.00

Figure S16: Ignoring competition can lead to an overestimation of the variant advantage. Estimated relative transmission advantage of the variant when it is introduced at A) 1% and B) 20% population immunity. Red line corresponds to the results obtained from the estimate_advantage function in EpiEstim, the green curve is obtained by taking the ratio of the variant and resident strain R_t, and the black line corresponds to the true transmission advantage of the variant. C-E) Results of the estimate_advantage EpiEstim function for different levels of transmission advantage and introduction times for the well-connected, heterogeneous and clustered networks. Entries in red correspond to underestimation in the variant advantage. See Suppl. Analysis for more details.

medRxiv preprint doi: https://doi.org/10.64898/2026.03.27.26349560; this version posted March 30, 2026. The copyright holder for this preprint (which was not certified by peer review) is the author/funder, who has granted medRxiv a license to display the preprint in perpetuity.
It is made available under a CC-BY 4.0 International license .

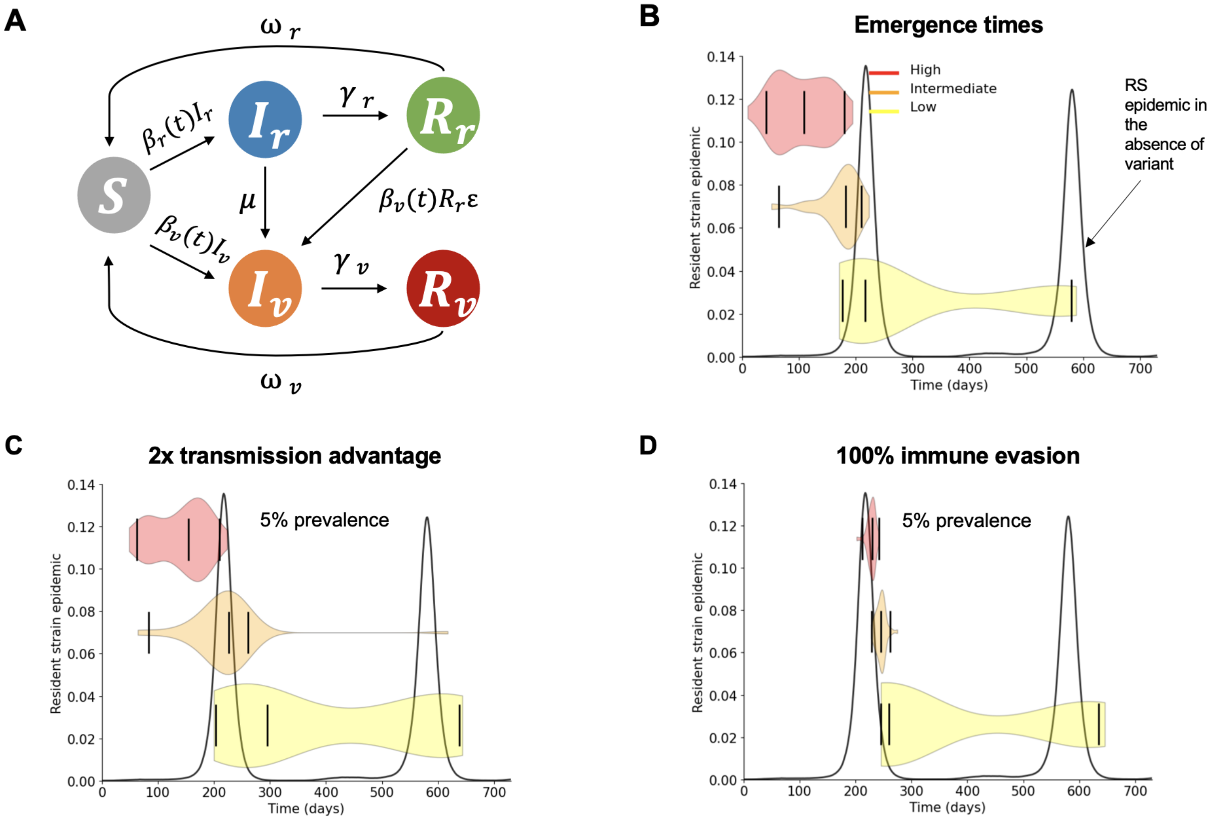

Figure S17: Model schematic and example variant detection times. A) Schematic of the two strain SIR-type model with a well-connected contact network consisting of individuals susceptible to infection (S), infected by the resident (I_r) or variant (I_v) strain, and recovered from infection by the resident (R_r) or variant (R_v) strain. For strain i, β_i is the per contact transmission rate which is time dependent and γ_i is the rate of recovery. Recovered individuals become susceptible at rate ω_i. The variant can be produced via mutation (rate μ) of the resident strain or imported into the population from an outside source. Variants with immune evasive properties ($0 < \epsilon <= 1$) can infect individuals recovered from the resident strain infection by a rate proportional to the strength of immune evasion ϵ. B) Variant emergence times for different rates of variant evolution. Times when the variant reaches 5% prevalence for a variant with C) transmission advantage and D) immune evasive properties . The resident strain epidemic in the absence of the variant is provided as a reference (black curve). Violin plots are marked with the median and the middle 95% quantile range. See Suppl. Analysis for more details.
